# The perfusion index measured by the pulse oximeter affects the agreement between ClearSight and the arterial catheter-based blood pressures: A prospective observational study

**DOI:** 10.1371/journal.pone.0219511

**Published:** 2019-07-10

**Authors:** Masashi Yokose, Takahiro Mihara, Masahiro Takaya, Takumi Yamamoto, Yusuke Saigusa, Shunsuke Takaki, Takahisa Goto

**Affiliations:** 1 Department of Anesthesiology and Critical Care Medicine, Yokohama City University Graduate School of Medicine, Yokohama, Japan; 2 Education and Training Department, Yokohama City University Hospital, YCU Centre for Novel and Exploratory Clinical Trials, Yokohama, Japan; 3 Department of Anesthesiology, Fujisawa City Hospital, Fujisawa, Kanagawa, Japan; 4 Department of Biostatistics, Yokohama City University Graduate School of Medicine, Yokohama, Japan; Cleveland Clinic, UNITED STATES

## Abstract

**Background:**

ClearSight is a noninvasive arterial blood pressure monitor, but it remains unknown whether it is affected by the state of perfusion to the fingers. We investigated whether the lower perfusion index (PI) measured with a pulse oximeter, which reflects finger perfusion, would affect the agreement between arterial pressures measured with ClearSight *versus* those obtained with an arterial catheter.

**Methods:**

Paired arterial pressure data (ClearSight and arterial catheter-based pressures) and PI values were prospectively obtained from 30 patients undergoing major abdominal surgery. The primary outcome was standard deviation (SD) of the bias (precision) of blood pressure between ClearSight and arterial catheter. The ratio of the adjusted SD of the bias between PI≤1 and PI>1 was calculated using the linear mixed-effects model. The secondary outcomes were the bias and the limits of agreement (LOA) between the two devices (repeated measures Bland-Altman analysis).

**Results:**

We analyzed 6312 paired data points. The adjusted SD of bias in PI ≤1 compared with those in PI >1 was 1.4-fold (95% confidence interval: 1.3- to 1.4-fold) for systolic arterial pressure, 1.5-fold (95% confidence interval: 1.3- to 1.6-fold) for diastolic arterial pressure, and 1.3-fold (95% confidence interval: 1.2- to 1.5-fold) for mean arterial pressure. The bias (LOA) were as follows: systolic arterial pressure in the PI ≤1 and PI >1 groups, -3.5 (-35.4 to 28.4) mmHg and 2.2 (-19.9 to 24.3) mmHg, respectively; diastolic arterial pressure in the PI ≤1 and PI >1 groups, 13.1 (-5.1 to 31.3) mmHg and 9.0, (-2.6 to 20.6) mmHg, respectively; and mean arterial pressure in the PI ≤1 and PI >1 groups, 8.7 (-11.3 to 28.7) mmHg and 7.6 (-6.2 to 21.3) mmHg, respectively.

**Conclusions:**

PI ≤1 was associated with a large SD of the bias between the devices. The PI value could be a real-time indicator of ClearSight precision.

## Introduction

Invasive arterial blood pressure measurement with an arterial catheter (IAP) is the gold standard for continuous and accurate monitoring of arterial blood pressure, but this technique carries the risk of complications such as arterial occlusion, pseudoaneurysm, local infection, or hematoma [1]. ClearSight (Edwards Lifesciences, Irvine, CA, USA), which consists of a cuff placed around a finger, is a noninvasive, continuous arterial blood pressure monitoring device. The pressure of the finger cuff is continuously adjusted so that the blood volume flowing in the finger arteries, and presumably the arterial diameter, is kept constant during the cardiac cycle (volume-clamp method). Based on the assumption that the transmural pressure is zero at this clamped finger artery diameter, the cuff pressure is considered equal to the finger arterial pressure. This “unloaded” state is checked by Physiocal calibration, which is automatically performed to maintain the appropriate diameter of the finger arteries [2]. Finally, a generalized algorithm developed by the manufacturer converts the finger arterial pressure waveform into the brachial arterial pressure [2].

The agreement between the arterial pressure values measured by ClearSight (APcs) and the arterial catheter-based blood pressures has been examined by several studies [3–6]. In patients undergoing cardiac surgery or those in the intensive care unit (ICU) after cardiac surgery, the APcs shows sufficiently good bias and precision to substitute for IAP. However, APcs is not reliable in critically ill patients or in those receiving vasopressors to maintain the blood pressure [7,8]. It has been speculated that these discrepancies may be caused by the reduced finger perfusion due to high peripheral vascular resistance, continuous vasopressor administration, and/or edema [7,8], but the evidence for these reasons is lacking.

We hypothesized that a lower peripheral perfusion would affect the agreement between the APcs and the IAP. As an indicator of peripheral perfusion, we chose the perfusion index (PI) measured by pulse oximetry, which is calculated as the ratio of the pulsatile infrared signal to the nonpulsatile infrared signal in the peripheral tissue [9,10]. The PI can be obtained continuously and noninvasively, and lower values indicate peripheral hypoperfusion or vasoconstriction.

The objective of this study was to assess whether PI affects the agreement between APcs and IAP.

## Materials and methods

### Study design and patients

This prospective observational study (Ethical Committee number: B161006003) was conducted according to the Declaration of Helsinki and was approved by the institutional ethics committee of the Yokohama City University Hospital, Yokohama, Japan on October 19, 2016. All patients gave written informed consent before inclusion. Our study was registered in the UMIN-CTR (registration number: UMIN000024824, principal investigator: Masashi Yokose, date: March 31, 2017) prior to patient enrollment. We prospectively enrolled consecutive patients aged ≥20 years who were scheduled to undergo an elective major hepatectomy (defined as liver resection ≥ 3 segments), pancreatoduodenectomy, or both under general anesthesia at the Yokohama City University Hospital, Yokohama, Japan, between April 2 and October 6, 2017. Radial artery catheter placement was planned for every patient because of clinical necessity. The exclusion criteria included pronounced disturbance of peripheral perfusion (Raynaud syndrome or peripheral artery occlusive disease), a known history of upper arm vascular surgery, and the presence or a known history of arterial fibrillation.

### Data measurement methods

The finger cuff of the ClearSight system was placed over the left middle phalanx of the third finger, ipsilateral to the radial artery catheter. The ‘Heart Reference System’ provided with the ClearSight device compensates for the hydrostatic difference between the finger and the heart. The Heart Reference System and the IAP transducer were zeroed at the midpoint of the right atrium as the reference level. IAP was monitored via a radial artery catheter (20G, SURFURO I.V. Catheter; TERUMO, Tokyo, Japan) and pressure transducer (FloTrac sensor; Edwards Lifesciences, Irvine, CA, USA) with standard low compliant tubing. The damping coefficient and natural frequency of the transducer were checked by the fast flush test at 1 minute before each measurement period. If inadequate damping was identified, we adjusted the condition of the arterial line (e.g., by exchanging the tube or checking the puncture site of the catheter). In cases where adjustment was not effective, we planned to exclude the data from the final analysis. The PI values were measured using a pulse oximeter (Masimo Radical 7; Masimo Corp., Irvine, CA). The pulse oximeter probe was attached to an ipsilateral finger not competing with the finger cuff of ClearSight. The bladder temperature measured by the urinary catheterization (Bardex Lubri-Sil I.C. Foley catheter; BARD, Covington, GA, USA) was recorded as the body temperature.

### Data acquisition

One measurement period was defined as 15 minutes. In the operating room, the anesthetists who were not involved in the data analysis planned to secure at least eight measurement periods from skin incision until skin suture when the hemodynamic condition was relatively stable. The study protocol did not require that these measurement periods be linked to any specific intraoperative events. In the ICU, at least two measurement periods were planned at every hour after arrival in the ICU per subject, so that the total number of measurement periods per subject would be at least 10. The systolic, diastolic, and mean arterial pressures obtained using ClearSight (SAP_CS_, DAP_CS_, and MAPcs, respectively) were recorded at 20-second intervals, and a set of three consecutive data points (obtained over 1 minute) were averaged to yield one datum. All APcs data were obtained at Physiocal intervals >30 beats that were considered to indicate stable and reliable pressure measurements [11]. IAP, heart rate, and body temperature were recorded at 1-minute intervals and stored on an anesthesia monitor (Life scope, DM-910P; Nihon Koden, Tokyo, Japan). PI values were recorded and stored at 10-second intervals on the pulse oximeter. The representative value of PI data obtained every minute was calculated by averaging six consecutive PI values. The PI values were blinded during data collection.

### Outcomes

In clinical decision-making, a large bias with a narrow limit of agreement (LOA) could be more useful than a small bias with a large LOA [12], because a large bias can be easily compensated for by calibration. Therefore, the primary outcome of our study was the standard deviation (SD) of the bias between arterial pressures measured with ClearSight *versus* those measured with an arterial catheter. The SD of the bias represents the precision of the ClearSight. We calculated the ratio of the adjusted SD of bias between PI ≤1 and >1 using the linear mixed-effects model. The secondary outcomes were the bias, LOA, percentage errors (PEs), and interclass correlation coefficients (ICCs) of these two devices.

### Sample size calculation

First, we defined the cutoff value of PI as 1 [13], which is described as indicative of hypoperfusion in the instruction manual and is also clinically acceptable. Then, we assumed that the number of blood pressure data points with PI ≤1 and those with PI >1 were equal. The SD of the bias between SAPcs and invasive systolic arterial pressure (ISAP) was assumed to be 8.4 mmHg [14]. The total number of data points (i.e., number of subjects multiplied by data points per subject) was determined to detect a 1.35-fold difference in the ratio of the SD of the bias between those with PI ≤1 and >1 (i.e., based on the assumption that a 30% increase in SD of the bias was clinically unacceptable). Assuming that at least 10 measurement periods could be secured per subject, the required number of patients was calculated by the F-test as 25 with a significance level of 0.05 and a power of 0.9. According to this calculation, we decided that the sample size was 30, and planned to obtain as many measurement periods as possible to increase the detection power.

### Statistical analysis

Values of APcs, IAP, and PI were imported into Microsoft Excel 2010 (Microsoft, Redmond, WA, USA). Then, the obvious irregular values caused by artefacts were identified by visual inspection.

The unadjusted bias and LOA (bias ± 1.96 SD) for APcs–IAP were calculated according to Bland-Altman analysis in overall data, data from PI ≤1, or data from PI >1, respectively. Bland-Altman analysis for repeated measurements was applied because we performed multiple observations per individual [15].

We employed the linear mixed-effects regression model to estimate the adjusted variances of the bias with PI ≤1 and PI >1 or the association between the covariate and the bias between two devices. In this model, the bias between two devices was used as the response variable. The heart rate, body temperature, binarized PI (≤1 / >1), and (APcs + IAP) / 2 (i.e., mean of measured values) were included as fixed effects. Subject was included as a random effect. The model allows the variances of the bias to differ depending on the binarized PI. The adjusted SD was calculated from this adjusted variance. The confidence intervals (CI) for ratio of adjusted SD of bias was calculated by the bootstrap method with 10,000 replications.

The PEs and ICCs were calculated using the datasets created as follows: the single mean values for IAP and APcs were calculated for each subject [i.e., the ISAP data sets contained a number of ISAP and SAPcs pairs equal to the total number of subjects, similarly for the invasive diastolic and mean arterial pressure (IDAP, IMAP, respectively) data sets]. The PE was calculated as [1.96 SD of bias / (mean value of IAP) * 100] to evaluate the interchangeability between the two methods [16]. In the ICC analysis, the agreement and consistency with 95% CI between IAP and APcs were calculated using two-way random reliability analysis [17,18].

All statistical analyses were performed using Microsoft Excel 2010 and R software version 3.3.2 (R foundation for Statistical Computing, Vienna, Austria). Data normality was assessed by the Kolmogorov-Smirnov test. All parametric data were presented as mean ± SD, and nonparametric data were presented as median [interquartile range, IQR] or number (percentage), as appropriate. The significance of the outcome was defined as a P value of less than 0.05 (two-tailed).

## Results

**Fig 1:**
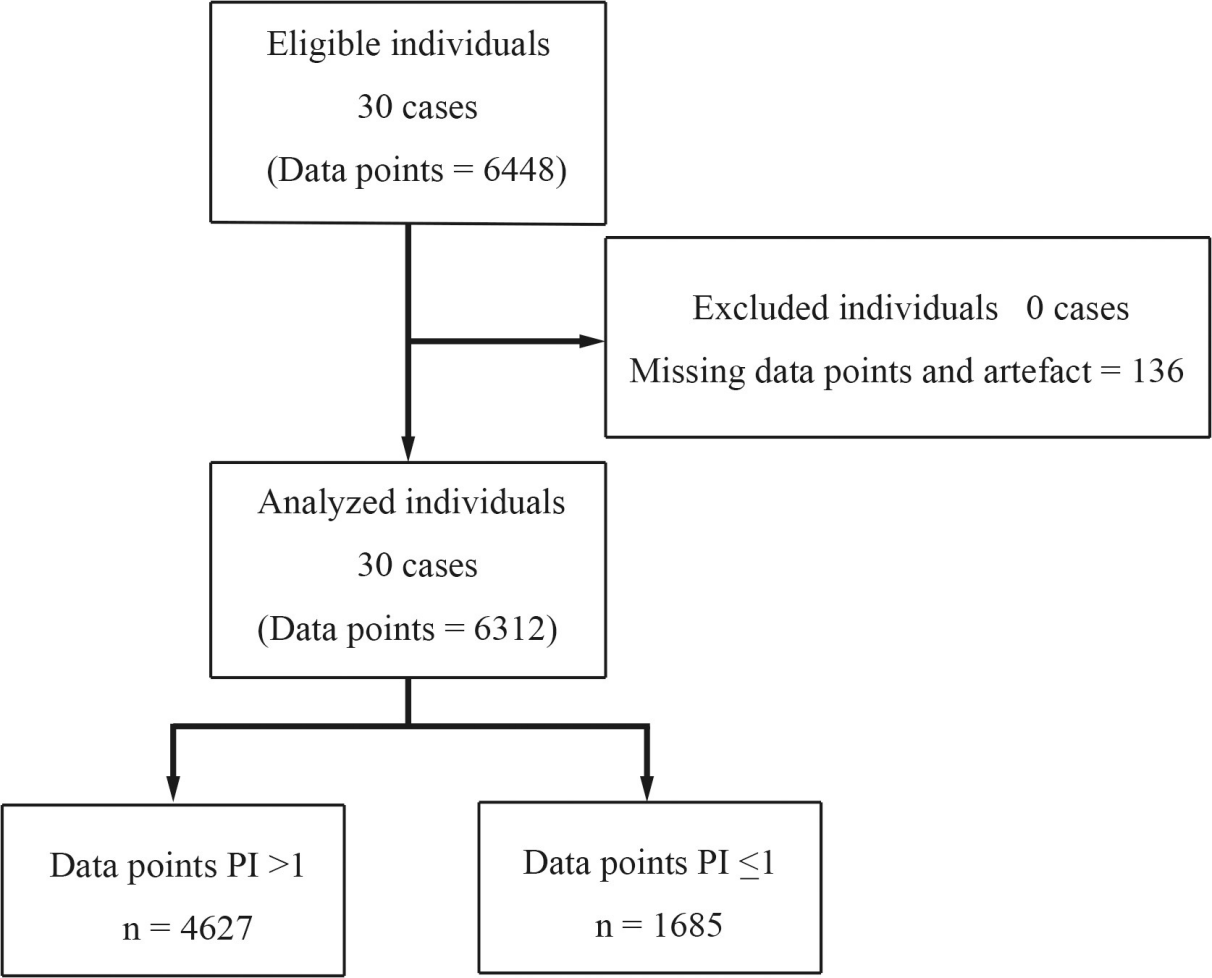
Patient flow diagram.

We collected 6448 paired data points from 30 patients. After excluding the missing data for IAP or APcs, and artefacts, 6312 valid, paired arterial pressure readings were compared. The number of data points for PI ≤1 was 1685. The median of paired data readings per subject was 211 [170–228] pairs. The ICU data could not be collected in 15 patients because of technical problems (ClearSight could not read the blood pressure signals) or operational problems (too large motion artefacts or the patients were not admitted to the ICU after surgery) (Fig 1).

**Fig 2:**
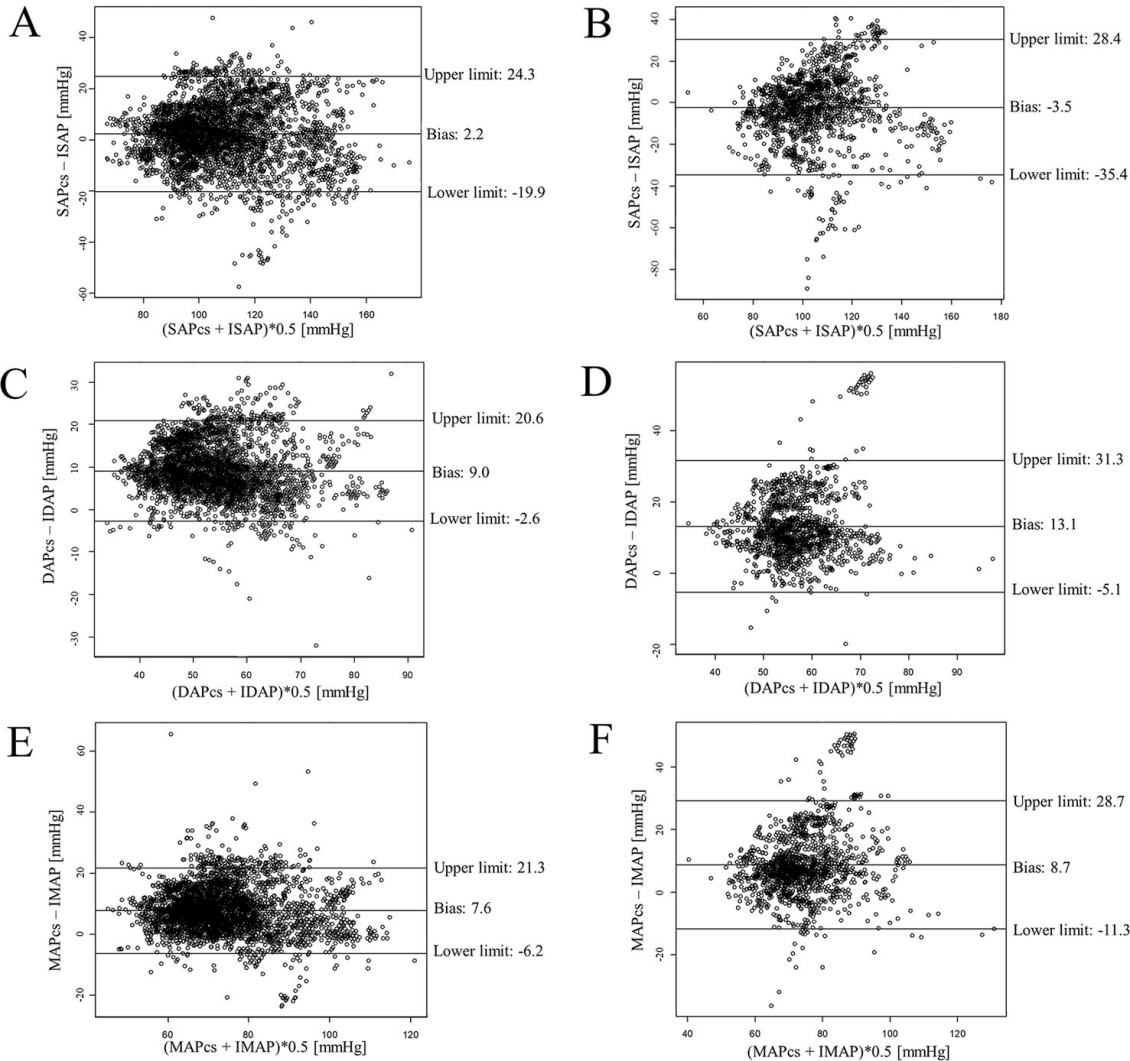
Bland-Altman plots with PI ≤1 and PI ≤1. Bland-Altman plot for systolic arterial pressure with PI ≤1 (A), systolic arterial pressure with PI >1 (B), diastolic arterial pressure with PI ≤1 (C), diastolic arterial pressure with PI >1 (D), mean arterial pressure with PI ≤1 (E), and mean arterial pressure with PI >1 (F). ISAP, invasive systolic arterial pressure; SAPcs, systolic arterial pressure measured by ClearSight; IDAP, invasive diastolic arterial pressure; DAPcs, diastolic arterial pressure measured by ClearSight; IMAP, invasive mean arterial pressure; MAPcs, mean arterial pressure measured by ClearSight; PI, perfusion index; Upper limit, upper 95% limit of agreement; Lower limit, lower 95% limit of agreement.

The patients’ characteristics, comorbidities, and anesthetic data are summarized in Table 1. In the Bland-Altman analysis of the overall data, SAPcs showed a small bias toward the ISAP, but had poor LOA (Table 2). DAPcs and MAPcs overestimated the IDAP and IMAP with large bias but showed relatively good LOAs compared to that in SAPcs (Table 2). The absolute values of unadjusted bias in the PI ≤1 group for the systolic, diastolic, and mean arterial pressure (SAP, DAP, and MAP, respectively) groups were larger than those in the PI >1 group (Table 2 and Fig 2). The 95% LOAs in the PI ≤1 group were wider than those in the PI >1 group (Table 2 and Fig 2).

**Table 1:** Patient characteristics.

Patients (n = 30)				
Age (years)				71 [67–76]
Sex, n (%)	Male			18 (60)
	Female			12 (40)
Height (cm)				162 (8.4)
Weight (kg)				56 (9.5)
BMI (kg m^-2^)				21.2 [20.3–22.7]
ASA-PS, n (%)		1		1 (3)
		2		27 (90)
		3		2 (7)
Comorbidities				
Hypertension, n (%)				9 (30)
Diabetes mellitus, n (%)				15 (50)
Anesthetic and operative data				
Systolic arterial pressure (mmHg)			Invasive	104 [94–116]
			Noninvasive	105 [91–119]
Diastolic arterial pressure (mmHg)			Invasive	50 [43–55]
			Noninvasive	60 [54–64]
Mean arterial pressure (mmHg)			Invasive	70 [62–75]
			Noninvasive	78 [70–84]
Hepatectomy, n (%)				14 (47)
Pancreatoduodenectomy, n (%)				12 (40)
Hepatectomy and pancreatoduodenectomy, n (%)				4 (13)
Anesthesia time (min)				837 [657–944]
Operation time (min)				747 [524–836]
Crystalloid volumes (mL)				4800 [4150–6188]
Colloid volumes (mL)				1750 [500–2113]
Transfusion volumes (mL)				140 [0–560]
Blood loss volumes (g)				1148 [568–1758]

All variables are expressed as mean (standard deviation), median [interquartile range], or number (percentage). ASA-PS, American Society of Anesthesiologists physical status; BMI, body mass index.

**Table 2:** Summary of the Bland-Altman analysis, percentage errors, and interclass correlation coefficients.

	Unadjusted Mean Bias (95% LOA) (mmHg)	Percentage error (95% CI) (%)	ICCs	
			Agreement (95% CI; P value)	Consistency (95% CI; P value)
SAP				
All data	0.7 (-24.6 to 26.0)	17.9 (17.5 to 18.4)	0.64 (0.24 to 0.83); P = 0.004	0.64 (0.24 to 0.83); P = 0.004
PI ≤1	-3.5 (-35.4 to 28.4)	22.2 (21.4 to 22.9)	0.52 (-0.20 to 0.81); P = 0.06	0.52 (-0.20 to 0.80); P = 0.06
PI >1	2.2 (-19.9 to 24.3)	15.8 (15.3 to 16.2)	0.77 (0.52 to 0.89); P<0.001	0.77 (0.52 to 0.89); P<0.001
DAP				
All data	10.1 (-3.8 to 24.0)	19.3 (18.8 to 19.8)	0.20 (-0.22 to 0.58); P = 0.279	0.53 (0.01 to 0.78); P = 0.024
PI ≤1	13.1 (-5.1 to 31.3)	22.0 (21.4 to 22.5)	(-0.14 to 0.26); P = 0.444	0.06 (-1.00 to 0.79); P = 0.445
PI >1	9.0 (-2.6 to 20.6)	15.7 (15.2 to 16.1)	0.41 (-0.19 to 0.78); P = 0.201	0.80 (0.57 to 0.90); P<0.001
MAP				
All data	7.9 (-7.6 to 23.3)	15.8 (15.4 to 16.2)	0.34 (-0.34 to 0.71); P = 0.197	0.59 (0.13 to 0.80); P = 0.01
PI ≤1	8.7 (-11.3 to 28.7)	19.3 (18.7 to 19.8)	0.28 (-0.36 to 0.68); P = 0.216	0.48 (-0.28 to 0.79); P = 0.076
PI >1	7.6 (-6.2 to 21.3)	13.8 (13.4 to 14.1)	0.54 (-0.30 to 0.84); P = 0.140	0.79 (0.56 to 0.90); P<0.001

Mean bias: mean of the differences (noninvasive–intra-arterial) between the 2 techniques. Limits of agreement (LOA): bias ± 1.96 SD of bias. Percentage error: 1.96 SD of bias / (invasive arterial pressure) * 100. Intraclass correlation coefficients (ICCs) analysis was calculated using the two-way random reliability analysis. CI, confidence interval; DAP, diastolic arterial pressure; MAP, mean arterial pressure; PI, perfusion index; SAP, systolic arterial pressure; SD, standard deviation.

In the linear mixed-effect regression model, the adjusted SD of bias in the PI ≤1 group for SAP, DAP, and MAP were significantly larger than those in the PI >1 group (Table 3). The absolute values of the adjusted mean differences were significantly reduced in SAP and DAP in the PI >1 group compared to those in the PI ≤1 group (Tables 3 and S1). The changes in the mean difference with each 1 degree of increase in body temperature were -1.5 (95% CI; -1.9 to -1.1) in SAP, -0.6 (95% CI; -0.8 to -0.4) in DAP, and -0.8 (95% CI; -1.0 to -0.5) in MAP. The influences of directionality on the biases of the other two factors were not consistent (S1 Table).

**Table 3:** The results of adjusted SD of bias and mean difference between noninvasive ClearSight arterial pressure and invasive arterial pressure measurements.

	Adjusted SD of bias (mmHg)		Ratio of the adjusted SD (95% CI)	Adjusted mean difference (mmHg)	
	PI ≤1	PI >1		Beta coefficient of PI >1 (95% CI)	P value
**Systolic Arterial Pressure**	9.5	7.0	1.4 (1.3 to 1.4)	3.6 (3.0 to 4.3)	<0.001
**Diastolic Arterial Pressure**	5.7	3.9	1.5 (1.3 to 1.6)	-2.3 (-2.7 to -2.0)	<0.001
**Mean Arterial Pressure**	6.3	4.8	1.3 (1.2 to 1.5)	-0.2 (-0.7 to 0.1)	0.20

Adjusted SD of bias, beta coefficient of PI >1, and P values were calculated by the linear mixed-effects regression model. Ratio of the adjusted SD: adjusted SD in PI ≤1/ adjusted SD in PI >1. 95% confidence interval (CI) was calculated by the bootstrap method with 10,000 replications. PI, perfusion index.

The PEs of SAPcs, DAPcs, and MAPcs in the PI ≤1 group were significantly higher than those in all the data or the PI >1 group (Table 2). In the PI >1 group, the agreement and consistency of ICCs for the SAPcs and the consistency for DAPcs and MAPcs showed relatively good correlations and 95% CI. In the PI ≤1 group, the overall agreement and consistency were poorer than those in the PI >1 group (Table 2).

## Discussion

We demonstrated that the PI ≤1 was associated with (1) reduced precision of APcs, indicated by a larger SD of bias, (2) increased biases between the two devices in SAP and DAP measurements, and (3) decreased interchangeability between the two devices. In the PI >1 group, the unadjusted mean difference of the biases between two devices and the LOAs were relatively better than those in the PI ≤1 group.

Our study suggests that the PI value could be used as a real-time indicator of the precision of the APcs. Specifically, we demonstrated that the precision of SAPcs, DAPcs, and MAPcs were reduced (i.e., the SD of bias increased) by 30% to 50% with PI ≤1 when compared to PI >1. In contrast, the changes in the bias between PI ≤1 and >1 were statistically significant but clinically marginal. We believe it is unlikely that our results were influenced by information bias, because PI values were blinded during the measuring period. In the clinical setting, lower precision is more difficult to compensate for than the larger bias. Therefore, our results may suggest the importance of maintaining PI >1 when using ClearSight.

The mechanism underlying the association between PI and precision or bias is unclear; however, it may be explained by the ClearSight algorithm, which converts the finger pressure signal to the brachial arterial pressure. The algorithm was constructed based on the data of the 53 subjects [2], including 15 healthy volunteers, seven elderly patients, 18 hypertensive patients, six subjects with therapy-resistant hypertension, and seven patients with hypertensive and arteriosclerotic vascular diseases. Notably, no perioperative subjects are included. Unlike the subjects included in the algorithm formulation, the patients undergoing surgery or in the ICU may experience a wider range of perfusion states caused by blood loss, insufficient fluid administration, hypothermia, and use of vasoconstrictors, to name but a few. Therefore, the pressure signal data used in the original algorithm may be insufficient to cover various clinical settings that the patients in our study were likely to have experienced. Increasing the variation of data samples may improve the precision of APcs even with low PI.

The bias, or LOAs in our results tended to be worse than those in previous reports [14,19], even when PI >1. The reason for this discrepancy is unclear but may include some methodological explanations as follows. First, some measurement errors or biological variations could not be excluded completely from our data. For example, in patients who had selected a relatively tight cuff for his or her finger, the mismatch of the cuff size due to the finger edema, which often occurs during a long surgery, might have caused the measurement error. Second, method comparison studies should preferably be performed in a hemodynamically stable situation, but some of our data might have been obtained in less stable situations. Because it would be difficult to predict the occurrence of the low PI value state beforehand in the perioperative setting, we randomly set the 15-min measurement periods at various stages of the operation and the postoperative ICU course. For the same reason, we did not plan to perform the “zero-zone” approach, which was the model used to account for the pathophysiological variations of reference arterial pressure [20–22]. Third, the cutoff value of PI, which was assumed as 1 to perform statistical analysis in this study, may not be appropriate. The threshold value of the low finger perfusion for PI measured by MASIMO Radical 7 has not been defined clearly, partly because PI shows skewed and widely variable values [10].

The PEs for APcs in the PI ≤1 group were significantly larger than those in the PI >1 group. The interchangeable threshold of the PE for APcs has not been reported. The interchangeable threshold of the PEs for CNAP (CNSystems Medizintechnik AG, Graz, Austria), which is a noninvasive arterial blood pressure monitor similar to ClearSight, were reported as 14.7% in SAP, 17.5% in DAP, and 18.7% in MAP [23]. The PEs for DAPcs (13.8%) and MAPcs (15.7%) in the PI >1 group were lower than these thresholds, whereas those in the PI ≤1 group were higher than these thresholds. We consider that MAPcs and DAPcs in the PI >1 situation are exchangeable for IAP, although further study will be needed to clarify the cutoff value of the PE in APcs.

### Limitations

Our study has several limitations. First, our study was performed at a single center, and the type of surgery was also limited. Therefore, our results may not apply to other conditions such as cardiac or emergency surgery, other age groups, or high-risk patients. Second, this is an observational study, so we could not conclude whether various interventions to change the PI (e.g., fluid administration, transfusion, uses of vasoactive drugs or anesthetics, etc.) similarly affect the precision of APcs. Third, we have only focused on blood pressure monitoring when discussing the exchangeability between ClearSight and the arterial catheter. However, the arterial catheter has many functions other than blood pressure monitoring (e.g., sampling of the blood, evaluation of preload). These aspects should be taken into consideration when clinicians decide whether the ClearSight can replace the arterial catheter in each individual clinical case.

## Conclusions

We found that PI ≤1 was associated with (1) decreasing the precision (i.e., increasing the SD of bias or LOA) of APcs, (2) increasing the biases between two devices in SAP and DAP, and (3) decreasing interchangeability between the two devices. The PI value could be used as a real-time indicator of the precision of the APcs. Further studies will be needed as to whether PI >1 is the best cutoff value for agreement between the two devices.

## Supporting information

S1 File Data of the patients included in this study. (XLSX)

S2 File STROBE checklist. (DOCX)

S3 File Study protocol in English. (DOCX)

S4 File Study protocol in Japanese. (DOCX)

S1 Table The results of the linear mixed-effects regression model for the mean difference between invasive arterial pressure and noninvasive ClearSight arterial pressure measurements. (DOCX)
