# Supplementary material for: The perfusion index measured by the pulse oximeter affects the agreement between ClearSight and the arterial catheter-based blood pressures: A prospective observational study
Source: PLoS One. 2019 Jul 10;14(7):e0219511. doi: 10.1371/journal.pone.0219511 (PMC6619788; doi:10.1371/journal.pone.0219511)
Supplement: S3 File — (DOCX) [file pone.0219511.s003.docx]

**Research Protocol**

**Evaluation of the index of accuracy at the time of hypoperfusion for noninvasive continuous arterial pressure monitoring: A method comparison study**

**Lead principal investigator: Masashi Yokose, Department of Anesthesiology and Critical Care Medicine, Yokohama City University Hospital**

**This protocol cannot be disclosed to a third party or used for any purpose other than the purpose of this research without the consent of the lead principal investigator.**

**Outline**

**0.1. Outline schema**

Patients undergoing an elective abdominal surgery (liver resection, pancreatoduodenectomy, or both) under general anesthesia

Informed consent

Registration

Data acquisition (operating room and intensive care unit)

Arterial blood pressure obtained by both noninvasive and invasive devices

Perfusion index (PI) derived from pulse oximeter

Estimating whether PI affects the agreement between the noninvasive and invasive arterial blood pressures

**0.2 Object and meaning**

ClearSight™ (Edwards Lifesciences Corporation, Irvine, CA USA) is a noninvasive monitor for measuring the arterial blood pressure, cardiac output, or other hemodynamic parameters. However, arterial blood pressure measured by the ClearSight is not reliable in critically ill patients or in those receiving vasopressors to maintain their blood pressure. It has been speculated that the unreliability may be due to reduced finger perfusion, but the evidence is lacking. The primary objective of this study is to assess whether the perfusion index (PI) affects the agreement between the arterial pressure measured by the ClearSight and the invasive arterial pressure.

**0.3. Selection of research subjects**

**Inclusion criteria**

(1) Aged ≥20 years

(2) Patients who are scheduled to undergo an elective major hepatectomy (defined as liver resection ≥ 3 segments), pancreatoduodenectomy, or both under general anesthesia.

(3) Cases with the indication for inserting an arterial catheter and for using the FloTrac system (Edwards Lifesciences).

(4) Patients with written informed consent for participation in this study.

**Exclusion criteria**

(1) Body weight <40 kg or >180 kg, body mass index >35 kg/m^2^.

(2) Patients with a pronounced disturbance of peripheral perfusion (Raynaud syndrome or peripheral artery occlusive disease).

(3) Patients with the presence of, or a known history of, arterial fibrillation.

(4) Patients with a known history of upper arm vascular surgery.

**0.4. Sample size and research period**

Estimated sample size: 30 cases

Research period: From the 19th of October 2016 to the 30th of September 2020

**0.5. Methods**

The arterial blood pressures measured by the ClearSight and the invasive arterial pressure are obtained in the operating room and the intensive care unit. The PIs are measured simultaneously with the measuring of the arterial pressures. In the analysis, all data are divided into two groups based on the PI value ≤1 / >1.

**Primary endpoint**

The standard deviation (SD) of the bias between arterial pressures measured with the ClearSight versus those measured with an arterial catheter.

**Analysis of the primary outcome**

Linear mixed-effects regression model (calculating the SD of the bias between the two devices)

Response variable: The mean differences between the two devices.

Covariate: Binarized PI (PI ≤1 and PI >1), heart rate, body temperature, the mean value of arterial pressure measured by the ClearSight (APcs), and the invasive arterial pressure.

Random effect: Subject

The model allows the variance of the bias to differ depending on the binarized PI.

Based on the results of this model, the ratio of the adjusted SD of bias is calculated.

If the PI value is determined to be an indicator of the agreement of the ClearSight and the invasive arterial pressure, it may be possible to judge the reliability of the APcs value in real time. Our study may beneficially contribute to patients by providing a minimally invasive solution.

0.6. **Lead principal investigator**

Masashi Yokose, Department of Anesthesiology and Critical Care Medicine, Yokohama City University Hospital

**1. Object and meaning**

ClearSight™ (Edwards Lifesciences Corporation, Irvine, CA USA) is a noninvasive monitor for measuring the arterial blood pressure, cardiac output (CO), or the other hemodynamic parameters such as stroke volume index (SVI), stroke volume variation (SVV), or systemic vascular resistance index (SVRI). With widespread use of the ClearSight, healthcare providers will be able to provide care in a less invasive manner.

The agreement between the arterial pressure values measured by the ClearSight (APcs) and the arterial catheter-based blood pressures (IAP) has been examined by several studies.[1-4] In patients undergoing cardiac surgery or those in the intensive care unit (ICU) after cardiac surgery, the APcs shows sufficiently good bias and precision to substitute for IAP. However, APcs is not reliable in critically ill patients or in those receiving vasopressors to maintain their blood pressure.[5-7] It has been speculated that the unreliability may be due to reduced finger perfusion from high peripheral vascular resistance, continuous vasopressor administration, and/or edema, but the evidence is lacking.

We hypothesized that lower peripheral perfusion would affect the agreement between the arterial APcs and the IAP. As an indicator of peripheral perfusion, we chose the perfusion index (PI) measured by pulse oximetry, which is calculated as the ratio of the pulsatile infrared signal to the non-pulsatile infrared signal in the peripheral tissue.[8] The PI can be obtained continuously and noninvasively, and lower values indicate peripheral hypoperfusion or vasoconstriction.

The primary objective of this study is to assess whether the PI affects the agreement between APcs and IAP. The secondary objective of this study is to assess whether the PI affects the agreement between cardiac output, SVI, SVV, or SVRI as measured by the ClearSight and those measured by the FloTrac system (Edwards Lifesciences Corporation, Irvine, CA USA). If the PI value is determined to be an indicator of the agreement of the ClearSight and the invasive arterial pressure, it may be possible to judge the reliability of the APcs value in real time. Our study may beneficially contribute to patients by providing a minimally invasive solution.

**2. Evidence of scientific rationality of research**

**2.1. Explanation of target disease**

The primary objective of this study is to assess whether the PI affects the agreement between the APcs and the IAP in the perioperative setting. If it can be determined that the PI is a real time index of the reliability of the ClearSight, this monitor may safely be used more widely in daily clinical practice.

Our study will require the participants to be using the ClearSight, an arterial catheter, and the FloTrac at same time. Therefore, we are targeting patients who are scheduled to undergo an elective major hepatectomy (defined as liver resection ≥ 3 segments), pancreatoduodenectomy, or both under general anesthesia, because the radial artery catheter placement and the use of the FloTrac system are planned for every one of these patients out of clinical necessity. The reasons are as follows: First, to avoid an unnecessary procedure for the subjects, and to reduce the burden on them as patients. Second, since intraoperative vasoconstriction occurs in operations that are large, invasive, and long, low PI values may be collected more frequently in these patients.

**2.2. Current status of treatment**

In our clinical setting, the placement of the arterial catheter and the use of the FloTrac are routinely performed for elective major hepatectomy, pancreatoduodenectomy, or both under general anesthesia. These operations are large, invasive, and long, and there is a need to manage the hemodynamics and the fluid balance strictly.

**2.3. Explanation of research**

In patients undergoing cardiac surgery or those in the ICU after cardiac surgery, the APcs shows sufficiently good bias and precision to substitute for IAP. However, APcs is not reliable in critically ill patients or in those receiving vasopressors to maintain their blood pressure. There is no report which has researched the real time index of the agreement of the IAP and the APcs with the ClearSight.

The protocol of this study is constructed based on the past similar studies which examined the agreement between the APcs and the IAP.

**3. Equipment information**

(1) EV1000 monitor, ClearSight finger cuff (Edwards Lifesciences Corporation, Irvine, CA USA)

This monitor system can noninvasively measure the arterial blood pressure by the cuff attached the subject’s finger without an invasive procedure such as the insertion of a catheter into the artery.

(2) FloTrac™ system (Edwards Lifesciences): Extracorporeal continuous cardiac output monitor

Based on the arterial pressure waveform from the transducer which is attached to a standard radial or femoral arterial catheter, this system can measure some hemodynamic parameters such as stroke volume (SV) or CO. This monitor will be used as a reference for comparison of the reliability of arterial blood pressures or other hemodynamic parameters measured by the ClearSight.

(3) Radical-7^®^ pulse oximeter (Masimo Corp., Irvine, CA USA)

This monitor can noninvasively measure pulse rate, peripheral oxygen saturation (SpO_2_), and the PI value, and is standard equipment for anesthesia management.

The pulse oximeter measures SpO_2_ by using the several wavelengths. The PI is calculated as the ratio of the pulsatile infrared signal to the non-pulsatile infrared signal in the peripheral tissue. Therefore, the PI value means the peripheral perfusion or vascular tone at the measurement site. A low PI value indicates poor peripheral perfusion.

**4. Diagnostic criteria and stage / disease type / pathology classification**

Not applicable

**5. Policy for the selection of study subjects**

**5.1. Inclusion criteria**

(1) Aged ≥20 years

(2) Patients who are scheduled to undergo an elective major hepatectomy (defined as liver resection ≥ 3 segments), pancreatoduodenectomy, or both under general anesthesia.

(3) Cases with the indication for inserting an arterial catheter, and for using the FloTrac system.

(4) Patients with written informed consent for participation in this study.

**5.2. Exclusion criteria**

(1) Body weight <40 kg or >180 kg, and body mass index >35 kg/m^2^.

(2) Patients with a pronounced disturbance of peripheral perfusion (Raynaud syndrome or peripheral artery occlusive disease).

(3) Patients with the presence of, or a known history of, arterial fibrillation.

(4) Patients with a known history of upper arm vascular surgery.

**6. Procedure of study participation and case registration / assignment**

**6.1.** This study will be registered with the University hospital Medical Information Network Clinical Trials Registry [UMIN-CTR (http://www.umin.ac.jp/ctr/index-j.htm)] for information disclosure.

**6.2. Facility registration**

Not applicable

**6.3. Case registration**

First, patients are confirmed to meet the inclusion criteria and to not meet the exclusion criteria. After patients give written informed consent, the patient information required to check eligibility will be entered on the study registration form. Before the final registration, the researcher who entered the registration form data and the other co-researchers will double-check each other to confirm.

**6.4. Allocation method**

Not applicable

**6.5. Blinding and Key Opening**

Not applicable

**7. Treatment plan**

**7.1. Study protocol**

(1) An arterial catheter is inserted into the left radial artery of each patient after the induction of general anesthesia.

(2) The finger cuff of the ClearSight system is placed over the left middle phalanx of the third finger, ipsilateral to the radial artery catheter.

(3) The pulse oximeter probe is attached to an ipsilateral finger not competing with the finger cuff of the ClearSight.

(4) All devices are calibrated.

(5) The management of intraoperative anesthesia is left to the anesthesiologist in charge. A specific anesthesia protocol is not defined. The data reported by the ClearSight are not considered in the perioperative management of the patient.

(6) The management of the patient in the ICU is left to the doctor in charge. A specific management protocol is not defined. The data reported by the ClearSight are not considered in the ICU management of the patient.

**Data acquisition**

**ClearSight:**

Systolic arterial pressure (SAPcs), diastolic arterial pressure (DAPcs), mean arterial pressure (MAPcs), CO, SV, and SVV data are recorded at 20-second intervals.

**FloTrac** **(arterial catheter):**

SAP, DAP, MAP, CO, SV, and SVI data are recorded at 20-second intervals.

**Radical-7 pulse oximeter:**

PI and SpO_2_ data are recorded at 10-second intervals.

**Anesthetic monitor in the operating room and the ICU:**

Heart rate, body temperature, and central venous pressure data are recorded at 1-minute intervals.

The systolic, diastolic, and mean arterial pressures, and the CO, SV, and SVI data obtained using the ClearSight and the FloTrac are recorded at 20-second intervals, and a set of three consecutive data points (obtained over 1 minute) are averaged to yield one datum. The IAP, heart rate, and body temperature are recorded at 1-minute intervals and stored on an anesthesia monitor. PI values are recorded and stored at 10-second intervals on the pulse oximeter. The representative value of PI data obtained every minute are calculated by averaging six consecutive PI values. No manual description of measured values is required, because these data are automatically stored in these measuring devices.

**Other data:**

(1) Edema, body temperature, history of hypertension, and dose of the vasopressors.

(2) The damping coefficient and natural frequency of the transducer are checked by the fast-flush test (square wave test) 1 minute before each measurement period.

(3) Time of the occurrence of artifact for APcs and IAP. These data are used for extracting the artifacts.

(4) Time information: anesthesia time, each measuring period, starting time of the operation, time of the specimen extraction, time of the intestinal anastomosis, and finishing time of the operation.

**End of the study**

The end of the study for each patient is defined as 24 hours after the start of data measurement or 4 hours after entering the ICU, whichever comes first.

**7.2. Dose and schedule change criteria**

The ClearSight shuts down after 8 hours of continuous monitoring to prevent blood stasis of the finger. The finger cuff is moved to another finger if continuous measurements of 8 hours or more will be performed.

**7.3. Discontinuance of protocol**

(1) Cases that cannot be measured due to technical problems of the devices.

(2) Cases in which it is difficult to continue the protocol due to the occurrence of adverse events.

(3) Cases found to be ineligible after the inclusion.

(4) For other reasons, if the doctor in charge determines that data collection cannot be continued.

**7.4. Combined treatment or supportive care**

Not applicable

**7.5. Post treatment**

Not applicable

**8. Burden, risk, and benefit on the study subject. Evaluation and measures to minimize the burden and risk**

**8.1. Definition of adverse event**

Information on serious complications regarding the use of the ClearSight have not been reported from the manufacturer. The occurrence rate of serious complications is considered to be rare, because the ClearSight is used based on the recommendation by the manufacturer. However, adverse events are defined as follows, based on all possibilities:

If the anesthesiologist in charge determines that data collection cannot be continued because of severe blood stasis at the cuff attachment site during the study period.

Furthermore, adverse events in this study are defined as unknown events that occur in relation to this study. Known adverse events and symptoms or complications related to the underlying disease or surgery are not included as adverse events of the study.

**8.2. Evaluate the adverse event**

Mild: No need for therapeutic intervention.

Severe: Immediate need for therapeutic intervention.

**8.3. Expected adverse events**

Blood stasis, swelling, ischemia, skin discoloration, numbness, and ache of the finger at the cuff attachment site of the ClearSight.

**8.4. Countermeasures for serious adverse events**

Not applicable

**8.5. Compensation for health damage**

Not applicable

**9. Observation, inspection, report items and schedule**

**9.1. Participation period of subjects**

Start of research study period: Time data collection begins after induction of the general anesthesia.

End of research study period: 4 hours after admission to the ICU or 24 hours after the start of the study period, whichever comes first.

**9.2. Observations, inspection, and information to report**

**9.2.1 Characteristics of subjects**

Height, weight, sex, American Society of Anesthesiologists (ASA) physical status, co-morbidity, and medical history.

**9.2.2 Perioperative data**

An arterial catheter is inserted into the left radial artery of each patient after the induction of general anesthesia. The finger cuff of the ClearSight system is placed over the left middle phalanx of the third finger, ipsilateral to the radial artery catheter. The pulse oximeter probe is attached to an ipsilateral finger not competing with the finger cuff of the ClearSight.

**ClearSight:**

Systolic arterial pressure (SAPcs), diastolic arterial pressure (DAPcs), mean arterial pressure (MAPcs), CO, SV, and SVV data are recorded at 20-second intervals.

**FloTrac (arterial catheter):**

SAP, DAP, MAP, CO, SV, and SVI data are recorded at 20-second intervals.

**Radical-7 pulse oximeter:**

PI and SpO_2_ data are recorded at 10-second intervals.

**Anesthetic monitor in the operating room and in the ICU:**

Heart rate, body temperature, and central venous pressure data are recorded in 1-minute intervals.

**Fast-flush test (square wave test):** Performed by the anesthesiologists both in the operating room and in the ICU and documented by filling-in the recording sheet.

**9.2.3 Other information to record**

**Time information:** anesthesia time, each measuring period, starting time of the operation, time of the specimen extraction, time of the intestinal anastomosis, and finishing time of the operation.

**The artifact of the IAP due to body movement or blood sampling**

**The information of the vasopressors**

These data are documented on the recording sheet by anesthesiologists in the operating room and the ICU, or nurses in the ICU.

**9.3. Measuring schedule**

One measurement period is defined as 15 minutes. In the operating room, anesthesiologists who are not involved in the data analysis plan for a two-hour operation that will result in at least 8 measurement periods from skin incision until skin suture when the hemodynamic condition is relatively stable. One hundred twenty data pairs will be obtained from one subject in the operating room. In the ICU, at least 2 measurement periods are planned per subject until 4 hours after arrival in the ICU, so that the total number of measurement periods per subject will be at least 10. We plan on obtaining 150 data points per subject, at least. However, these are just target numbers. It is possible to increase or decrease the measurement periods depending on the actual operation situation or the time of admission to the ICU.

**10. Definition of the endpoints**

**10.1 Primary endpoint**

The standard deviation (SD) of the bias between arterial pressures measured with the ClearSight versus those measured with an arterial catheter.

**10.2 Secondary endpoint**

(1) The beta coefficient of the bias using the linear mixed-effects model between arterial pressures measured with the ClearSight versus those measured with an arterial catheter.

(2) The percentage error of the arterial pressure.

(3) Interclass correlation coefficients of the arterial pressure.

(4) The beta coefficient of the CO, SV, and SVV calculated by the linear mixed-effects regression model.

**11. Statistical analysis**

**11.1 Sample size**

**Estimated sample size before starting the recruitment: 30 cases**

**Grounds:** First, the cut-off value of the PI is defined as 1 (described in the pulse oximeter manual as a measure of hypoperfusion and clinical acceptability). Second, it is assumed that the acquirable number of data points of the PI ≤ 1 and those points of the PI > 1 are equal. The bias of the arterial blood pressures between the two devices is assumed to be 8.4 mm Hg based on a past study.[9] The total number of data points is determined to detect a 1.35-fold difference in the ratio of the SD of the bias between those with a PI ≤1 and >1. Assuming that at least 10 measurement periods can be secured per subject, the sample size is calculated by an *F*-test to be 25 with an *α* error of 0.05 and a 1–*β* of 0.9. We are planning for a sample size of 30 cases in anticipation of possible dropout. According to the Association for the Advancement of Medical Instrumentation (AAMI) SP10.2002, a minimum of 15 subjects and 10 readings per subject should be reported for the proof of accuracy for noninvasive blood pressure (NIBP) devices. This criterion has been applied in the past in similar studies about the accuracy of the noninvasive arterial pressure monitor.[10] In comparison to the above, our sample size is considered to be roughly reasonable. The measurement period should be set as evenly as possible during the operation.

**11.2. Analysis sets**

Statistical analysis is performed using all cases for which the data collection is properly performed.

**11.3. Methods and items**

**11.3.1** **Analysis of the primary outcome**

**Linear mixed-effects regression model**

**(calculating the SD of the bias between the two devices)**

Response variable: The mean differences between the two devices.

Covariate: Binarized PI (PI ≤1 and PI >1), heart rate, body temperature, and the mean values of the APcs and the IAP.

Random effect: Subject

The model allows the variances of the bias to differ depending on the binarized PI.

Based on the results of this model, the ratio of the adjusted SD of bias is calculated.

**Bland-Altman analysis for repeated measurements**

After all data are classified into the two groups based on the PI ≤1 and PI >1, Bland-Altman analysis will be performed in each group to calculate the bias, precision, and the 95% limits of agreement.

**11.3.2 Analysis of the secondary outcome**

(1) The bias between the two devices is calculated by the linear mixed-effects regression model for the primary outcome.

(2), (3) For the evaluation of the interchangeability, the percentage error and interclass correlation coefficients with 95% confidence interval between the two devices are calculated.

(4) Linear mixed-effects regression model

The dependent values: The mean difference of CO, SV, and SVV between the two devices

The independent value: PI value

The random effect: Subject

The CO, SV, and SVV data pairs of the two devices are divided into two groups based on PI value 1. Bland-Altman analysis for repeated measurements will be performed in each group, and bias, precision, and 95% limits of agreement are calculated.

**11.4 Interim analysis**

Not applicable

**12. Case report forms**

Case report data are reported on the form that includes the patient characteristic data, perioperative data, and case registration. The co-researcher will submit this form to the lead principal investigator after completing the form.

**13. Efficacy and safety evaluation committee**

Not applicable

**14. Ethical matter**

**14.1 Ethical regulations**

We adhere to the ethical principles based on the Declaration of Helsinki. This study is conducted according to the Ethical Guidelines for Medical and Health Research Involving Human Subjects established by the Ministry of Health, Labor and Welfare of Japan. All research is conducted according this protocol. If any deviation or change from this protocol occurs for unavoidable or medical reasons, the reason will be described on the case report form. If these deviations or changes may be considered severe with regard to the study management, the research manager should consider the discontinuation of the study or the modification of the protocol.

**14.2 Handling of personal information**

When personal information is handled, a symbol unrelated to the personal information of the research subject is attached and anonymized so that the individual cannot be identified at a glance. The correspondence table is kept at the independent computer which is not connected to the hospital LAN or to the internet at the Department of Anesthesiology, Yokohama City University Hospital. The management of personal information follows the procedure manual about the handling of such personal information in clinical research at Yokohama City University Hospital. At viewing of the raw data of the study or a written consent form, and publication of research results, we give due consideration to the privacy protection of the subjects. Personal information obtained in this research is not released to third parties.

**15. Procedures for receiving informed consent**

Researchers explain to the subjects about the following information by using the document which was approved by the ethical committee. After confirming that the patient fully understands the information, written consent for participation is obtained.

If the consent is not obtained from the patient in the event that they cannot confirm their intention to participate, we will obtain the consent of the representative of the patient. If the subject recovers to a state conducive for giving consent while participating in this study, the researchers will explain to subject again and obtain written consent.

1. Approvals received to conduct the research
2. The duration of the study and the name of the lead principal investigator
3. Objective and meaning
4. Methods and protocol
5. Inclusion and exclusion criteria
6. Burden, risk, and benefit for participation in this research
7. Withdrawal from the study
8. Subjects will not be disadvantaged
9. Other treatments (Not applicable for this study.)
10. Viewing authority for data or information of subjects
11. Handling of personal information
12. Storage and disposal of the data
13. Conflict of interest
14. Consultation from research subjects and their related persons
15. Expense for this study
16. Medical care after this research (Not applicable for this study.)
17. Policy for incidental findings
18. Compensation for health damage
19. Possibility of reusing the data obtained in this study for a future study
20. Browsing data and information by researchers
21. Publication of research results
22. Policy for the intellectual property rights

**15.1 Consultation from research subjects and their related persons**

If a subject requests to view data or information of this study, the researchers will provide the information that can be disclosed as appropriate, except for personal information of the researchers, and non-replicable parts such as protection of intellectual property rights of the researchers.

**16. Storage and disposal of the data**

Written consents, case report forms, and paper media for data recording are stored on a lockable shelf. Electronic media are recorded on a PC requiring the password for login. This PC is deployed in a lockable room. These documents and data related to this study (i.e. the documentation for ethical committee, case report forms, or consent forms) are to be kept on a lockable shelf until 5 years after end of this study or 3 years after result publication. After this period, these data will be completely deleted.

**17. Research found, conflict of interest, personal income for this study**

**17.1. Financial sources and relationships**

The EV1000 monitor is rented for a fee by Yokohama City University Hospital. Consumable goods are purchased by the Department of Anesthesiology, Yokohama City University Hospital. This study is conducted as medical research. It does not provide benefits to, or for the convenience of, a particular company or organization.

**17.2. Expense for this clinical study**

All medical expenses related to this study are calculated based on the National Health Insurance of Japan. These expenses shall be paid by the subject.

**17.3. Compensation for health damage**

The use of the EV1000 monitor in daily clinical practice has been approved by the Ministry of Health, Labor and Welfare, Japan. Therefore, there is no financial compensation for health damages.

**18. Modifying or changing of the study protocol**

This study protocol is modified or changed by the research manager, as necessary. Regardless of the severity of the change, the research manager will submit the modified protocol and the documentation that describes the revised content and the reason for the change to the chairman of the research institution.

1. The case of a major change:

(i.e., changes in eligibility criteria, treatment plans, endpoints, expected adverse events).

Ask the ethics committee for deliberation and obtain approval.

1. The case of a minor change:

(i.e., a protocol change that does not increase the subject's risk, or changes not related to the primary endpoint).

This requires the approval of the main researchers involved in the plan and reporting to the ethics committee.

**19. Contents and method of report to the director of the institute**

**19.1 Progress report of research**

The progress report of research will be described once a year.

**19.2 End of the research**

At the end of this study, the summary of results will be reported by the research manager to the director of the institute.

**19.3 Early termination of research**

If the research is discontinued or interrupted, the research manager will report the situation to the director of the research institute. Specifically:

(1) If the safety of the study is questioned, or if it is judged that the significance of the study continuing no longer exists.

(2) If it is judged that the successful execution of the study is difficult (i.e. significant delay in case registration, or many protocol deviation events).

**20. Management of the documentation for this study**

The documentation and data related to this study (i.e. the documentation for the ethical committee, the case report forms, or the consent forms) are securely stored on a lockable shelf until 5 years after end of this study or 3 years after resulting publication.

**21. Information disclosure of this study**

The results of the research will be presented at an international congress or in an academic journal. The first author is defined as lead principal investigator. Co-authors will be determined by discussions among those involved in the study.

**22. Monitoring and audit**

Not applicable

**23. Organizational structure of this study**

**23.1 Research manager**

Masashi Yokose

Department of Anesthesiology and Critical Care Medicine

**23.2 Research office**

Not applicable

**23.3 Other research institutes**

Not applicable; Single center study

**23.4. Outsourcing work**

Not applicable

**23.5 Clinical statistician**

Not applicable

**23.6 Case registration and data management**

Not applicable

**24. References**

1. Martina JR, Westerhof BE, van Goudoever J, et al. Noninvasive continuous arterial blood pressure monitoring with Nexfin(R). *Anesthesiology* 2012;116:1092–103.
2. Ameloot K, Van De Vijver K, Van Regenmortel N, et al. Validation study of Nexfin(R) continuous non-invasive blood pressure monitoring in critically ill adult patients. *Minerva Anestesiol* 2014;80:1294–301.
3. Fischer MO, Avram R, Cârjaliu I, et al. Non-invasive continuous arterial pressure and cardiac index monitoring with Nexfin after cardiac surgery. *Br J Anthesth* 2012;109:514–21.
4. Hofhuizen C, Lansdorp B, Hoeven JG van der, et al. Validation of noninvasive pulse contour cardiac output using finger arterial pressure in cardiac surgery patients requiring fluid therapy. *J Crit Care* 2014;29:161–5.
5. Hohn A, Defosse JM, Becker S, et al. Non-invasive continuous arterial pressure monitoring with Nexfin does not sufficiently replace invasive measurements in critically ill patients. *Br J Anaesth* 2013;111:178–84.
6. Monnet X, Picard F, Lidzborski E, et al. The estimation of cardiac output by the Nexfin device is of poor reliability for tracking the effects of a fluid challenge. *Crit Care* 2012;16:R212.
7. Stover JF, Stocker R, Lenherr R, et al. Noninvasive cardiac output and blood pressure monitoring cannot replace an invasive monitoring system in critically ill patients. *BMC Anesthesiol* 2009;9:6.
8. Lima AP, Beelen P, Bakker J. Use of a peripheral perfusion index derived from the pulse oximetry signal as a noninvasive indicator of perfusion. *Crit Care Med* 2002;30:1210–3.
9. Kim S-H, Lilot M, Sidhu KS, et al. Accuracy and precision of continuous noninvasive arterial pressure monitoring compared with invasive arterial pressure: a systematic review and meta-analysis. *Anesthesiology* 2014;120:1080–97.
10. Smolle K-H, Schmid M, Prettenthaler H, et al. The accuracy of the CNAP(R) device compared with invasive radial artery measurements for providing continuous noninvasive arterial blood pressure readings at a medical intensive care unit: a method-comparison study. *Anesth Analg* 2015;121:1508–16.
