# Supplementary material for: The perfusion index measured by the pulse oximeter affects the agreement between ClearSight and the arterial catheter-based blood pressures: A prospective observational study
Source: PLoS One. 2019 Jul 10;14(7):e0219511. doi: 10.1371/journal.pone.0219511 (PMC6619788; doi:10.1371/journal.pone.0219511)
Supplement: S4 File — (DOCX) [file pone.0219511.s004.docx]

非侵襲的・連続的動脈圧測定モニターの

低灌流時の正確性の指標についての検討

：方法比較試験

研究代表者：［所属］横浜市立大学附属病院　麻酔科 ［名前］横瀬　真志

2016年8月31日　第1.1版

2017年9月30日 第1.2版

2018年8月28日　第2.0版

2019年3月18日　第3.0版

機密情報に関する注意

本プロトコルは機密情報であり、本研究の関係者に提供されるものです。

被験者への説明の場合を除き、研究代表者の同意なしに第三者に開示または本研究の目的以外に使用することはできません。

機密情報に関する注意

本臨床試験実施計画書は、機密情報であり、本試験に参加する試験実施医療機関、研究責任医師、担当医師、試験協力者、倫理審査委員会、効果安全性評価委員会、データセンター、等の試験関係者に対して提供されるものです。

本試験実施計画書は、被験者に対して本試験の内容を説明する場合を除き、主任研究者、副主任研究者の文書による同意なしに、いかなる第三者にも開示または本試験の目的以外に利用することはできません。

機密情報に関する注意

本臨床試験実施計画書は、機密情報であり、本試験に参加する試験実施医療機関、研究責任医師、担当医師、試験協力者、倫理審査委員会、効果安全性評価委員会、データセンター、等の試験関係者に対して提供されるものです。

本試験実施計画書は、被験者に対して本試験の内容を説明する場合を除き、主任研究者、副主任研究者の文書による同意なしに、いかなる第三者にも開示または本試験の目的以外に利用することはできません。

機密情報に関する注意

本臨床試験実施計画書は、機密情報であり、本試験に参加する試験実施医療機関、研究責任医師、担当医師、試験協力者、倫理審査委員会、効果安全性評価委員会、データセンター、等の試験関係者に対して提供されるものです。

本試験実施計画書は、被験者に対して本試験の内容を説明する場合を除き、主任研究者、副主任研究者の文書による同意なしに、いかなる第三者にも開示または本試験の目的以外に利用することはできません。

機密情報に関する注意

本臨床試験実施計画書は、機密情報であり、本試験に参加する試験実施医療機関、研究責任医師、担当医師、試験協力者、倫理審査委員会、効果安全性評価委員会、データセンター、等の試験関係者に対して提供されるものです。

本試験実施計画書は、被験者に対して本試験の内容を説明する場合を除き、主任研究者、副主任研究者の文書による同意なしに、いかなる第三者にも開示または本試験の目的以外に利用することはできません。

機密情報に関する注意

本臨床試験実施計画書は、機密情報であり、本試験に参加する試験実施医療機関、研究責任医師、担当医師、試験協力者、倫理審査委員会、効果安全性評価委員会、データセンター、等の試験関係者に対して提供されるものです。

本試験実施計画書は、被験者に対して本試験の内容を説明する場合を除き、主任研究者、副主任研究者の文書による同意なしに、いかなる第三者にも開示または本試験の目的以外に利用することはできません。

目次

0. 概要

0.1シェーマ

0.2目的及び意義

0.3研究対象者の選定方針

0.4予定登録数と研究期間

0.5研究の方法

0.6研究代表者と問い合わせ先

1. 目的及び意義

2. 研究の科学的合理性の根拠

2.1対象疾患の説明

2.2治療の現状

2.3 研究の説明

3. 薬物／機器情報

3.1 薬物情報

3.2 機器情報

4. 診断基準と病期・病型・病態分類

5. 研究対象者の選定方針

5.1 選択規準

5.2 除外規準

6. 研究参加の手続きと症例登録・割付

6.1 研究登録

6.2　施設の研究参加登録

6.3　症例登録及び割付手順

6.4　割付方法と割付調整因子

6.5　盲検下とキーオープン

7. 治療計画

7.1 プロトコル治療

7.2 用量・スケジュール変更規準

7.3 プロトコル治療の中止

7.4 併用治療・支持療法

7.5 後治療

通常の診療を超える医療行為を伴う研究の場合には、研究対象者への研究実施後における医療の提供に関する対応

8. 研究対象者に生じる負担並びに予測されるリスク及び利益、これらの総合的評価、当該負担及びリスクを最小化する対策

8.1 有害事象の定義

8.2 有害事象の評価と報告

8.3 予期される有害事象等

8.4 重篤な有害事象が発生した際の対応(侵襲（軽微な侵襲を除く。）を伴う研究の場合

8.5　健康被害に対する補償の有無及びその内容(侵襲を伴う研究の場合)

9. 観察・検査・報告項目とスケジユール

9.1 被験者の研究参加期間

9.2 観察・検査項目及び報告すべき情報

9.3 観察・検査・報告スケジュール

10. 評価項目（エンドポイント）の定義

10.1主要評価項目(プライマリーエンドポイント)

10.2副次評価項目（セカンダリーエンドポイント）

11. 統計学的事項

11.1 目標登録症例数と設定根拠

11.2 解析対象集団

11.3 解析項目・方法

11.4 中間解析

12. 症例報告書の記入と提出

13. 効果安全性評価委員会

14. 倫理的事項

14.1 遵守すべき諸規則

14.2　個人情報等の取扱い (匿名化する場合にはその方法を含む)

15. インフォームド・コンセントを受ける手続

15.1　研究対象者等及びその関係者からの相談等への対応

15.2　代諾者等からインフォームド・コンセントを受ける場合

15.3　インフォームド・アセントを得る場合

15.4　研究対象者に緊急かつ明白な生命の危機が生じている状況における研究の実施

15.5　研究対象者に係る研究結果（偶発的所見を含む。）の取扱い

16　試料・情報の保管及び廃棄

16.1 試料・情報のバイオバンクとしての利用

17. 研究の資金源等、研究機関の研究に係る利益相反及び個人の収益等、研究者等の研究に係る利益相反に関する状況

17.1 資金源及び財政上の関係

17.2 臨床研究に関する費用

(研究対象者等に経済的負担又は謝礼がある場合には、その旨及びその内容)

17.3 健康被害に対する補償

18. 研究実施計画書の改変更

19. 研究機関の長への報告内容及び方法

19.1 研究の終了

19.2 研究の早期中止

20. 記録の保存

21. 研究に関する情報公開の方法

22. モニタリング及び監査

23. 研究の実施体制

23.1研究代表者

23.2研究事務局

23.3参加施設及び施設の研究責任者

23.4 委託業務の内容及び委託先の監督方法

23.5臨床統計家

23.6症例登録割付及びデータマネジメント

24．文献

25．付録

概要

- 1. シェーマ

肝切除術・膵頭十二指腸切除術を受ける20歳以上の患者

説明・同意取得

登録

データ収集（手術中・術後集中治療室）

非侵襲的連続動脈圧・観血的動脈圧の測定

脈波灌流指数（＝末梢灌流の指標）の測定

脈波灌流指数の値で分けた末梢低灌流時と通常時の

非侵襲的連続動脈圧の

測定精度の比較

- 1. 目的及び意義

非侵襲的・連続的動脈圧測定モニター（ClearSight System^®^：以下クリアサイト）は動脈への侵襲的な手技を必要とせずに連続的に動脈圧を測定できる機器であり、手術中や集中治療領域での使用が拡大すればより低侵襲な医療の提供が可能となる。しかしながら、末梢血管緊張による低灌流状態ではクリアサイトで測定される動脈圧とこれまでの測定方法との間の誤差は大きくなることが明らかとなっている。低灌流状態によって生じるクリアサイトの測定精度の誤差が許容範囲かどうかを反映する簡便な指標は現在のところ報告されていない。今回の研究では，非侵襲的に測定可能な血管緊張性の指標である脈波灌流指数（Perfusion index: PI）がクリアサイトの測定精度の許容範囲を示す指標となるかどうかについて全身麻酔手術患者を対象に検討する。

- 1. 研究対象者の選定方針

**選択基準**

以下のすべての項目を満たす患者を対象とする。

1. 予定された手術に対して全身麻酔を受ける20歳以上の患者
2. 肝臓摘出術・膵頭十二指腸切除術を受ける患者
3. （2）の手術に際して麻酔管理上、観血的動脈圧測定と低侵襲性心拍出モニター(FloTrac^®^センサー)が必要な患者
4. 本研究の参加に関して同意が文書で得られる患者

**除外基準**

以下のいずれか一つ以上の項目を満たす場合は研究対象外とする。

1. 体重<40 or >180 kg, BMI >35 kg m^-2^
2. レイノー症候群やその類縁疾患の既往がある患者
3. 心房細動がある患者（副次項目のデータ収集に影響を与えるため）
4. 上腕の血管手術の既往がある患者
   1. 予定登録数と研究期間

予定登録数は30名とする。期間は2016年10月19日から2020年9月30日までとする。

- 1. 研究の方法

・方法比較研究：侵襲を伴わない介入研究

・手術中・術後ICUにおいてクリアサイトと従来からの観血的動脈圧を用いて動脈圧を測定する。また、末梢灌流指標として脈波変動指数（PI）を測定する。PIの値は血管収縮状態を反映して逐次、増減する（血管収縮状態では減少し・拡張状態では高値となる）。このPI値の減少に伴ってクリサイトで測定した動脈血圧の誤差が大きくなるのかを統計学的に比較し解析する。

・評価項目： PI＞１のバイアス^＊^の標準偏差（＝precision）に対するPI≦１バイアスの標準偏差の比　　（^＊^バイアス＝二つの測定方法間の平均値差）

（一般的に低灌流状態のめやすとされているPI＝１をカットオフポイントとした）

・統計解析方法：線形混合効果モデル

PI≤1、PI> 1それぞれのデータ群における2つの測定方法間のバイアスの標準偏差を推定する

従属変数：測定法間の1分間の平均値差

共変量：PI値（PI≦１/>1の二値）、心拍数、体温、クリアサイト動脈圧と観血的動脈圧の平均

ランダム効果項：個人

このモデルで得られたバイアスの標準偏差をもとに主要評価項目であるバイアスの標準偏差の比を計算する。

　　　・A Bland–Altman analysis for repeated measurements

PI＝１を基準に両測定動脈圧データペアを2群に分け、それぞれの群で繰り返し測定を考慮に入れたBland-Altman解析を行い、バイアス及び95%limits of agreementを図示する。

　・研究の意義： PI値が測定誤差の許容範囲を示す指標となる事が明らかとなれば、PI値を参考にすることで、クリアサイトの測定値の信頼性をリアルタイムに判断でき、観血的動脈圧測定の代替がクリアサイトで可能となる臨床的な場面が広がる可能性がある。

- 1. 研究代表者と問い合わせ先

所属：麻酔科　　　　氏名：横瀬　真志 　　　　　問い合わせ先：045-787-2918

1. 目的及び意義

非侵襲的・連続的動脈圧測定モニター（ClearSight System^®^ [ Edwards Lifesciences©]：以下クリアサイト）は非侵襲的に動脈血圧や心拍出量cardiac output: CO（それに伴って計算される一回拍出量係数[Stroke volume index: SVI]や一回拍出量変動［Stroke volume variation: SVV］、全身血管抵抗［Systemic vascular resistance index: SVRI］などの循環動態パラメータ）を動脈への侵襲的な手技（観血的動脈圧測定）を必要とせずに連続測定できる医療機器であり、手術中の麻酔管理や集中治療領域での使用が拡大すればより低侵襲な医療の提供が可能となる。

これまでクリアサイトで計測された動脈圧は麻酔中や集中治療患者を対象とした研究においてこれまでの動脈圧測定のスタンダードである観血的動脈圧を比較対象として、精度・信頼性・交換可能性について検討がなされている^1-4^。これらの報告によると心臓手術中やその術後の集中治療室での治療という条件のもとでは観血的動脈圧を代替できる精度があることが明らかとなった。一方で集中治療室に入室するような重症患者や血圧維持目的に血管収縮薬を使用している患者ではクリアサイトによる動脈圧測定の正確性が低下することが言われている^5-7^。原因としては種々の原因に伴う血管収縮状態と説明されている。血管収縮状態でのクリアサイトの測定精度の誤差が臨床的に許容範囲内にあるかどうかをリアルタイムに反映する簡便な指標は現在のところ報告されていない。

脈波灌流指数（Perfusion Index:以下 PI）はMASIMO社のパルスオキシメータ（動脈血酸素飽和度と脈拍数を非侵襲的・連続的に測定できる装置）で計測される数値である。パルスオキシメータは数種類の波長の光を用いることで酸素飽和度を計測している。測定の過程で吸光される光は，動的成分（拍動性信号：吸収される光の量が変動する拍動部分）と静的成分（非拍動性信号：拍動しない血管，皮膚，組織といった一定量の光が吸収される非拍動部分）から成り立っていて，これらの比率をPIと定義としている^8^．PIはその数値が大きいほど動的成分が多いことを示し，非侵襲的かつ連続的に測定部位（多くの場合は指）での末梢灌流状態や血管緊張性を表している。PI値が高い場合には灌流状態が良い事を示し、逆に低い場合には灌流状態が悪いことを表す。PIは手術中にパルスオキシメータに付随した標準モニターとして連続的に測定することが可能であり、実際に当院の手術室においても全症例で計測されている。われわれはクリアサイトによる動脈圧測定精度に影響を与える血管収縮の程度をPI値が反映できる可能性があるとの仮説を立てた。

今回の研究では，非侵襲的に測定可能な血管緊張性の指標であるPIがクリアサイトの測定精度の許容範囲を示す指標となるかについて全身麻酔手術患者を対象に検討する。主要評価項目としての動脈圧に関しては従来からのゴールデンスタンダードである侵襲的動脈圧との測定誤差を評価する。副次的評価項目としての心拍出量とそれに伴った循環動態パラメータ（SVI・SVV・SVRI）については体外式連続心拍出量測定用センサー（低侵襲性心拍出モニター）であるFloTrac®（Edwards Lifescience ©）（以下フロートラック）で計測された値との間で比較検討する。

PI値がクリアサイトの測定誤差の許容範囲を示す指標となる事が明らかとなれば、PI値を参考にすることで（例えばカットオフ値以下のPI値の時にはクリアサイトの値は信頼できない等）、クリアサイトの測定値の信頼性をリアルタイムに判断でき、観血的動脈圧測定の代替がクリアサイトで可能となる臨床的な場面が広がることに貢献できる可能性がある。

1. 研究の科学的合理性の根拠
   1. [対象疾患の説明等]

今回の研究目的は，非侵襲的に測定可能な血管緊張性の指標であるPIがクリアサイトの測定精度の許容範囲を示す指標となるかについて全身麻酔手術患者を対象に検討する事である。クリアサイトの血管収縮状態での信頼度をPI値がリアルタイムに示すことができれば、これまで以上にクリアサイトを安全に使用できる範囲が広がる可能性がある。

クリアサイトで測定される動脈圧を比較するためには標準的な連続的動脈圧測定法である、観血的動脈圧測定と比較することが必要である。そのため、麻酔管理上で観血的動脈圧測定が必要となる手術を対象とすることが研究上の患者負担を減らすうえで望ましい。さらに、手術中の血管収縮は大きな侵襲の長時間手術で生じるため、このような条件に合致した症例を選択することが必要である。また副次的評価項目を検討するためには1．で述べたようにフロートラックとの比較が必要であり、麻酔管理上で必要となるような手術を対象とすることが必要である。これらの条件に合致する手術を検討した結果本研究では予定された肝切除術または、膵頭十二指腸切除術を受ける全身麻酔患者を研究対象とする。

- 1. [治療の現状等]

肝切除術および膵頭十二指腸切除術を受ける患者の標準的な麻酔方法は、全身麻酔と硬膜外麻酔を併用した方法である。肝切除術は大きな侵襲の手術であり、手術に伴う出血や門脈遮断・下大静脈遮断などの手術手技によって循環動態の大きな変動が余儀なくされる。また、膵頭十二指腸切除術も侵襲の大きな長時間手術であり、出血や血行再建などの手術手技によって循環動態の変動が大きく、水分バランス管理が重要な手術である。そのため、当院ではすべての症例で観血的動脈圧測定、フロートラックおよび中心静脈カテーテルの挿入によって、血行動態管理を行っている。

- 1. [研究の説明等] ・方法等

これまでの先行研究では心臓手術中、およびその術後の集中治療室において観血的動脈圧測定している患者を対象にクリアサイトを併用して動脈血圧を測定し、クリアサイトの正確性を検討している。これらによると血管収縮状態や血管収縮薬を使用している状態ではクリアサイトの正確性に問題があることが明らかとなっている。クリアサイトの信頼性をリアルタイムで評価できる指標を検討した研究はこれまでのところ報告はない。

研究の方法自体は過去に類似した比較研究が多数あり、それらの方法を踏襲する形でデザインする。

1. 薬物／機器情報

　研究対象機器：

（１）EV1000モニター及び クリアサイトフィンガーカフ

（ともにEdwards　Lifescience©承認番号：22300BZX00363）指先に装着したカフ

（マンシェット）によって非侵襲的に動脈圧を計測できるシステム。最大の特長は手の指先に専用のカフを着けるのみで、従来のような動脈へのカテーテル挿入を行わなくとも動脈圧が連続的に測定できる点である。

（２）FloTracセンサー^®^（Edwards Lifescience^©^）：体外式連続心拍出量測定用センサー

観血的動脈ラインに接続し得られた動脈圧波形情報に基づいて、一回拍出量や心拍出量などの各種パラメータを連続的かつ低侵襲に測定できるシステム。クリアサイトとの比較の基準としてフロートラックを用いて動脈圧・心拍出量や一回拍出量といったパラメータの正確性を評価する。

（3）Radical-7^®^パルスオキシメータ（MASIMO^©^）

脈拍数・動脈血酸素飽和度およびPIを非侵襲的・連続的に測定する装置。標準的な麻酔中の患者モニタデバイスである。

パルスオキシメータは数種類の波長の光を用いることで酸素飽和度を計測している。測定の過程で吸光される光は，動的成分（拍動性信号：吸収される光の量が変動する拍動部分）と静的成分（非拍動性信号：拍動しない血管，皮膚，組織といった一定量の光が吸収される非拍動部分）から成り立っていて，これらの比率をPIと定義としている^8^．PIはその数値が大きいほど動的成分が多いことを示し，非侵襲的かつ連続的に測定部位（多くの場合は指）での末梢灌流状態や血管緊張性を表している。PI値が高い場合には灌流状態が良い事を示し、逆に低い場合には灌流状態が悪いことを表す。

1. 診断基準と病期・病型・病態分類

特定の疾患の診断や病期・病型・病態分類は考慮する必要がないため本研究では該当しない

1. 研究対象者の選定方針
   1. 選択基準

以下のすべての項目を満たす患者を対象とする

1. 20歳以上の患者
2. 肝臓摘出術もしくは膵頭十二指腸切除術を全身麻酔下に受ける患者
3. 観血的動脈圧測定を行い、かつフロートラックを使用する患者
4. 本研究の参加に関して同意が文書で得られる患者
   1. 除外基準

以下のいずれか一つ以上の項目を満たす場合に除外とする

1. 体重<40 or >180 kg, and BMI >35 kg/m^2^

（著しい肥満やるい痩患者ではデータの信用性が低下するとの報告がある。）

1. レイノー症候群やその類縁疾患の既往がある患者
2. 心房細動がある患者　（副次項目のデータ収集に影響を与えるため）
3. 上腕の血管手術の既往のある患者
4. 研究参加の手続きと症例登録・割付
   1. 研究登録

本研究はUMIN臨床試験登録システム［UMIN Clinical Trials Registry (UMIN-CTR) <http://www.umin.ac.jp/ctr/index-j.htm>］に登録し、情報公開する。

- 1. 施設の研究参加登録

本研究に該当しない。

- 1. 症例登録及び割付手順

対象患者が選択基準(5.1.)をみたし、除外基準(5.2.)に該当しないことを確認し、同意を取得した後、選択基準・除外基準が記載されている症例登録表に必要な患者情報を記載する。登録票を記載した医師とそれ以外の研究分担者とが相互にダブルチェックを行い選択基準・除外基準ともに適切であることを確認した後に本登録とする。

- 1. 割付方法と割付調整因子

ランダム化比較試験ではないため該当しない。

- 1. 盲検化とキーオープン

クリアサイトで収集されたデータは麻酔管理には使用しない。盲検化の必要はないため該当しない。

1. 治療計画
   1. **研究プロトコル**

・全身麻酔導入後に観血的動脈圧ラインを挿入する。挿入部位は左橈骨動脈を第一選択とする。

・観血的動脈圧ライン挿入部位と同側の指にクリアサイトのフィンガーカフを装着する。

・通常の麻酔モニターとして使用している経皮的酸素飽和度測定用のプローベを観血的動脈圧ライン挿入部位と同側の指に装着する。

・すべての機器のキャリブレーションが終了し測定が可能となった状態で研究開始とする。

・麻酔方法は担当麻酔科医師に一任し、もっとも適切であると判断した麻酔管理を行い、特にプロトコルを定めない。また、研究用に使用するクリアサイトのモニター表示される測定値は麻酔管理方針の判断基準としての使用はしない。

●クリアサイトでの収集データ

・収縮期血圧、拡張期血圧、平均血圧、心拍出量、一回心拍出量、一回心拍出量変動

データは20秒単位で本体内に記録される。

●フロートラックでの収集データ

・収縮期血圧、拡張期血圧、平均血圧、心拍出量、一回心拍出量、一回心拍出量変動

データは20秒単位で本体内に記録される。

●Radical-7　パルスオキシメータのデータ

・脈波灌流指数（PI）、酸素飽和度

10秒単位にパルスオキシメータ本体へデータが記録される。

●手術室内・集中治療室内の生体モニター情報

・心拍数・体温（深部体温・皮膚温）・中心静脈圧

1分単位で生体モニター本体へデータが記録される。

・これらのモニターからデータは自動的に測定機器本体及び生体モニターへストアされるため、測定値の用手的な記載は不要である。

→上記3つのデータの取り扱いについて

・データ測定期間とポイントの定義とポイント数について

一回のデータ測定期間は連続した15分間とする。

二つの測定方法間の動脈血圧の差とPI値は1分間の平均値を1ポイントとして定義する。

よって、一回のデータ測定期間において15ポイントのデータ収集を行う。

●その他、手術中に抽出する情報

（主にクリアサイトによる動脈圧や心拍出量等に影響を与える可能性のある因子）

＊浮腫・体温（特に皮膚温）、高血圧の既往（動脈硬化）、ノルアドレナリンの持続投与量

＊その他の記載項目

・動脈圧ラインの正確性の評価：rapid flash test（a square wave test）、動脈圧波形の見た目

（動脈圧波形の正確性を判定するために行われる一般的なテスト。短時間ヘパリン生食で圧ライン内をフラッシュした時の圧波形の形をみて、動脈ラインの「なまり」を評価する。「なまり」があると動脈圧を過小評価するため、このテストを担当麻酔科医師が施行し、結果を記録用紙へ記載する。）

・その他測定時の動脈圧値のアーチファクトが認められた時刻

　　　各種アーチファクト（例　クリアサイトの測定不可の状態・観血的動脈圧モニターの値が何らかの原因で表示されていない状態：例えば動脈圧ラインからの採血や動脈圧ラインの「なまり」）を記録用紙に記載し、後のデータ解析時に除外する。

・手術開始時刻/肝切除開始時刻（肝切除術）/検体摘出時刻/手術終了時刻

・手術終了後は集中治療室への移動のため各種測定を一旦中止する。集中治療室へ移動後より各モニターのキャリブレーション実施後にデータ収集を再開する。集中治療室では到着後1時間後から4時間後までの間に1時間ごとに15分の測定期間を設け（最低2回）データ収集を行う。その間のアーチファクトについては手術中と同様に所定の用紙に看護師・もしくは医師が記載する。

・集中治療室での治療方法については、特にプロトコルを定めず、各担当医師の裁量に一任する。また、クリアサイトのデータは治療方針の決定には用いない。

・手術開始前の測定開始から24時間経過した時点もしくは集中治療室に入室後4時間経過するまでのうち早い期間で研究終了とする。

- 1. 用量・スケジュール変更基準

クリアサイトフィンガーカフは指のうっ血を防ぐために8時間で自動的に測定が停止される。8時間以上の連続測定が行われる場合にはフィンガーカフを違う指に付け替えて測定を継続する。

- 1. プロトコル治療の中止

以下の基準に該当した場合、施設の研究責任医師または担当医師はプロトコル治療を中止する。

1. 機器の不具合で計測ができなかった症例
2. 有害事象の発現により医師が試験治療の継続困難と判断した場合
3. 登録後、不適格症例であることが判明した場合
4. その他の理由で、担当医師がデータ収集の継続ができないと判断した場合
   1. 併用治療・支持療法

併用治療・支持療法については本研究では該当しない。

- - 1. 併用禁止治療

本研究では該当しない

- - 1. 併用可能治療

本研究では該当しない

- 1. 後治療

本研究では該当しない。

1. 研究対象者に生じる負担並びに予測されるリスク及び利益、これらの総合的評価、当該負担及びリスクを最小化する対策
   1. 有害事象の定義

製造販売元からの情報では現在までに重篤な合併症の報告はない。メーカー推奨の使用方法の範疇で使用する本研究においては、発生頻度は低いと考えられるが考えられうる可能性から有害事象について以下のように定義した。

―クリアサイトフィンガーカフ装着部位のうっ血が著明となり、担当麻酔科医師が測定継続困難と判断した場合を有害事象とする。

　　　本研究における有害事象とは、本研究に関連して発生した有害事象のうち未知の物と定義し、既知のものや原疾患・手術に関連して発生した症状・合併症は本研究における有害事象には含めない。

- 1. 有害事象の評価

うっ血の程度によって以下のように評価する。

軽度（治療介入の必要がない、経過観察のみで改善が認められる見込みである）

重度（直ちに薬物治療などの治療介入が必要となる可能性）

- 1. 予期される有害事象等

クリアサイト装着部のうっ血・腫脹、虚血、皮膚の変色（青または赤への変色）、指先のしびれ・うずき

- 1. 重篤な有害事象が発生した際の対応

(侵襲（軽微な侵襲を除く。）を伴う研究の場合)

　　　本研究には該当しない。

8.5.健康被害に対する補償の有無及びその内容

(侵襲を伴う研究の場合)

　　　本研究には該当しない。

1. 観察・検査・報告項目とスケジユール
   1. 被験者の試験参加期間

研究開始：全身麻酔開始後にデータを計測開始した時刻

研究終了：集中治療室入室後4時間経過後もしくは研究開始から24時間後までのうちどちらか早い時刻

- 1. 観察・検査項目及び報告すべき情報
     1. 被験者背景

登録時調査する。登録前1ヶ月以内のデータを用いる。

身長、体重、性別、American society of Anesthesiologists Physical Status、合併症、既往歴

- - 1. 臨床所見（術中・術後データ）

全身麻酔導入後に観血的動脈圧ラインを挿入する。観血的動脈圧ライン挿入部位と同側の指にクリアサイトのフィンガーカフと経皮的酸素飽和度測定用のプローベを装着する。

●クリアサイトでの収集データ

・収縮期血圧、拡張期血圧、平均血圧、心拍出量、一回心拍出量、一回心拍出量変動

データは20秒単位で本体内に記録される

●フロートラックでの収集データ

・収縮期血圧、拡張期血圧、平均血圧、心拍出量、一回心拍出量、一回心拍出量変動

データは20秒単位で本体内に記録される。

●Radical-7　パルスオキシメータのデータ

・PI、酸素飽和度

10秒単位にパルスオキシメータ本体へデータが記録される。

→上記3つのデータの取り扱いについて

・データ測定期間とポイントの定義とポイント数について

一回のデータ測定期間は連続した15分間とする。

二つの測定方法間の動脈血圧の差とPI値は1分間の平均値を1ポイントとして定義する。

よって、一回のデータ測定期間において15ポイントのデータ収集を行う。

その他、収集するデータ

　これらは主に背景の情報として収集する

●手術室内・集中治療室内の生体モニター情報

・心拍数・体温（深部体温・皮膚温）・中心静脈圧

1分単位で生体モニター本体へデータが記録される。

●観血的動脈圧波形情報

・Square flash test：動脈圧波形の正確性を判定するために行われる一般的なテスト。短時間ヘパリン生食で圧ライン内をフラッシュした時の圧波形の形をみて、動脈ラインの「なまり」を評価する。「なまり」があると動脈圧を過小評価するため、このテストを担当麻酔科医師が施行し、結果を記録用紙へ記載する。

- - 1. 手術中・集中治療室で記載する情報（その他の情報）

●手術中

・時刻の記載

（測定開始、手術開始時間、肝切除開始（肝切除術の場合）、検体摘出、腸管再建終了時刻（膵頭十二指腸切除術の場合）手術終了時刻）

●手術中・集中治療室

・観血的動脈圧波形のアーチファクト（主には採血時や体動によるアーチファクト）

・血管収縮薬（ノルアドレナリン）の使用量と使用時間

→調査票を作成し麻酔科医や集中治療医・看護師がこれらの情報を記載する。

- 1. 観察・検査・報告スケジュール

術中から術後集中治療室にかけて循環動態パラメータとパルスオキシメータのデータを連続的に測定し研究終了までデータを取集する。

・データ測定期間とポイントの定義とポイント数について

一回のデータ測定期間は連続した15分間とする。

二つの測定方法間の動脈血圧の差は1分間の平均値を1ポイントとして定義する。

よって、一回のデータ測定期間において15ポイントのデータ収集を行う。

・手術室内

１肝切除術

麻酔導入後－手術開始前（データ測定期間1回15分：15ポイント×1）

手術開始後－肝切除開始前（データ測定期間2回各15分ずつ：15ポイント×2）

肝切除開始―検体摘出（データ測定期間3回各15分ずつ：15ポイント×3）

検体摘出―手術終了（データ測定期間2回15分：15ポイント×2）

データ測定期間：最低8回　総ポイント数8回×15ポイント＝120ポイント

30人のデータで総ポイント数は最低3600ポイントとなる

2膵頭十二指腸切除術

麻酔導入後―手術開始前（データ測定期間1回15分：15ポイント×1）

手術開始後―検体摘出（データ測定期間回3回15分：15ポイント×3）

検体摘出―腸管再建終了（データ測定期間２回15分：15ポイント×2）

腸管再建終了―手術終了（データ測定期間2回15分：15ポイント×2）

データ測定期間：最低8回　総ポイント数8回×15ポイント＝120ポイント

30人のデータで総ポイント数は最低3600ポイントとなる

・集中治療室内

①入室後60-120分、②入室後120-180分、③入室後180-240分の3つの期間のあいだに15分間データ収集期間を最低2回設ける。

15分間のデータ収集期間の間に得られたデータは1分間の平均値を1ポイントとして定義し、15分間で15ポイント、合計2回の測定を行い、1症例あたり30ポイント、30症例で900ポイントの計測を計画する。

　　以上より、１症例あたり120＋30＝150ポイント計測できる予定である。

これらのポイント数はあくまで目標数であり、実際の手術進行や集中治療室入室の時間帯などの理由によるポイント数の増減は許容する。

1. 評価項目（エンドポイント）の定義
   1. 主要評価項目（プライマリーエンドポイント）

11統計学的事項に示す線形混合モデルにおけるPI＞１のバイアスの標準偏差（＝precision）に対するPI≦１バイアスの標準偏差（＝precision）の比

- 1. 副次評価項目（セカンダリーエンドポイント）

（1）線形混合モデルにおける二つの測定方法間のバイアスに対するPIのβ係数

（2）2つの測定方法間のパーセント誤差（すべてのデータ、PI≦1、PI＞1に分けて計算）

（3）2つの測定方法間の級内相関係数（すべてのデータ、PI≦1、PI＞1に分けて計算）

（4）2つ測定法間（クリアサイトとフロートラック）の心拍出量、一回心拍出量、一回心拍出量変動の平均値の差を従属変数、PI値を独立変数、個人を変量切片とした線形混合モデルにおけるPIのβ係数

1. 統計学的事項
   1. 目標登録症例数と設定根拠

目標登録症例数： 30例

根拠：PIのカットオフ値を1（パルスオキシメータ―の取扱説明書に低灌流の目安として記載があり、臨床的にも許容可能）と定義し、取得できるPI≦1の血圧データポイントとPI＞1の血圧データポイント数が等しいと仮定した。 2つの測定方法間の間のバイアスの標準偏差（＝precision）は、過去の研究^９^から8.4 mmHgと仮定した。バイアスの標準偏差の30％の増加は臨床的に許容できないとの仮定のもとに、PI＞1に対するPI≦１のバイアスの標準偏差の比が1.35倍の差を検出できうるデータポイントの総数を計算した。一人の対象者あたり10回の測定期間が取得可能な場合、αエラーを0.05、検出力を0.9としF検定を用いる場合25人が必要と計算された。これを参考としたうえで研究の実行可能性を考慮し、若干の解析除外例を見込んで30例を目標登録症例数とする。Smolleら（2015）^10^の類似する非侵襲的連続動脈圧測定モニターの有用性についての研究報告を参照するとAssociation for the Advancement of Medical Institutionの SP10というクライテリアでは最低15人の測定で１人から10ポイントのデータ収集が必要と記載されており、計算された数字はおおよそ妥当な範囲であると考えられる。手術の進行に合わせて手術開始前・肝臓切除開始前（膵頭十二指腸切除術の場合には検体摘出前後）・肝臓摘出後～手術終了といった区分に分け、偏りすぎないようにデータ採取を行う。

- 1. 解析対象集団

解析対象集団の区別は特には行わず、適切にデータ採取が行われた全症例を用いて統計解析を行う。

- 1. 解析項目・方法
     1. 主要評価項目の解析

・線形混合効果モデル

　PI≤1、PI> 1それぞれのデータ群において2つの測定方法間のバイアスの標準偏差の推定する

従属変数：測定法間の1分間の平均値の差

共変量：PI値（PI≦１/>1の二値）、心拍数、体温、クリアサイト動脈圧と観血的動脈圧の平均

ランダム効果項：個人

得られたPI≦１、PI＞１それぞれのバイアスの標準偏差をもとに、バイアスの標準偏差比を計算する。

・A Bland–Altman analysis for repeated measurements

PI＝１を基準に両測定動脈圧データのペアを2群に分け、それぞれの群について繰り返し測定を考慮に入れたBland-Altman解析を行い、バイアス、95％一致限界などの計算値を図示する。

- - 1. 副次評価項目の解析

（1）2つの測定方法間のバイアスと共変量との関連性の推定

主要評価項目で用いた線形混合効果モデルを用いて、2つの測定方法間のバイアスとPIを含む共変数との関連性を調べる。関連の強さはPIのβ値およびP値で評価する。

（2）（3）２つの測定方法間のパーセント誤差および級内相関係数

すべてのデータ、PI≦1、およびPI＞1に分けて計算する。95%信頼区間を計算し、PI値の影響を比較する。

（4）2つ測定法間（クリアサイトとフロートラック）の心拍出量、一回心拍出量、一回心拍出量変動の平均値の差を従属変数、PI値を独立変数、個人を変量切片とした線形混合モデルを用いる。関連の強さはそれぞれのβ値で評価する。PI＝１を基準に2つの測定方法の心拍出量、一回拍出量、一回心拍出量変動データのペアを2群に分け、それぞれの群について繰り返し測定を考慮に入れたBland-Altman解析を行い、バイアス、95％一致限界などの計算値を図示する。

- 1. 中間解析

研究の内容から、現時点では中間解析を行う必要性はないと考えている。

1. 症例報告書の記入と提出

手術中・術後に記載するデータ記載用紙、患者背景記入用紙、症例登録書、症例報告書は一つの用紙で兼用することで運用する。研究期間終了後、必要事項記載後は速やかに研究代表者へ提出する。

1. 効果安全性評価委員会

本研究においては該当しない。

1. 倫理的事項
   1. 遵守すべき諸規則

本研究は、ヘルシンキ宣言に基づいた倫理原則を遵守し、「人を対象とする医学系研究に関する倫理指針（平成27年4月1日施行）」に従って実施する。

担当医師は、本プロトコルを遵守して麻酔・術後管理を実施する。被験者の緊急の危険を回避するため、その他医療上やむを得ない理由によりプロトコルから逸脱あるいは変更をした場合は、その理由を症例報告書に記載する。

また、その内容が本研究の継続に重大となる場合は、研究代表者が研究中止、研究計画の変更などを検討する（0．研究実施計画書の変更）。

14.2. 個人情報等の取扱い

本研究では、情報を取り扱う際には研究対象者の個人情報とは無関係の記号を付し、一見して個人が特定できないように匿名化する。対応表はパスワードを設定し、横浜市立大学麻酔科学教室内の院内 LAN やインターネットに接続されていない独立したコンピューター端末で管理する。対応表の管理を含め、研究対象者の個人情報は、横浜市立大学附属病院の臨床研究に関する個人情報等の取扱いに関する手順書に従う（管理責任者：横瀬　真志）。研究の実施に関する原データ類および被験者の同意書等の直接閲覧、並びに研究成果の公表においては、被験者患者のプライバシー保護に十分配慮する。本研究で得られた個人情報は、第三者へ漏洩しない。

1. インフォームド・コンセントを受ける手続

麻酔担当医師は、患者が研究に参加する前に、施設の倫理委員会で承認された同意・説明文書を用いて以下の事項を十分に説明する。患者が内容を十分理解したことを確認した後、参加の同意を文書により取得する。

なお、患者が文書同意の能力を欠く等により、本人からの同意を得ることが困難な場合には、代諾者からの同意を得ることとするが、この場合、同意に関する記録とともに代諾者と患者との関係を示す記録を残す。また、患者が本研究参加中に同意可能な状態に回復した場合、担当医師はあらためて患者に同意説明を行い、患者本人から文書により同意を取得する。

１．研究の許可を受けていること

２．研究期間及び研究責任者

３．研究の目的及び意義

４．研究の方法及び期間

５．研究対象者として選定された理由

６．負担並びに予測されるリスク及び利益

７．撤回できること

８．不利益を受けないこと

９．他の治療方法について

10．資料の閲覧

11．個人情報等の取り扱い

12．試料・情報の保管及び廃棄の方法

13．利益相反（起こり得る利害の衝突）

14．研究対象者等及びその関係者からの相談

15．費用について

16．研究実施後の医療提供

　　　手術に伴う合併症や原疾患に対する予後などには影響を与えない研究のため、該当しない

17．偶発的所見の取り扱い

　　　診断的介入を行わない研究であるため、本研究においては該当しない

18．健康被害への補償

19．将来の研究の可能性

将来の研究の可能性については現時点ではないため該当しない。

20．関係者が試料・情報を閲覧すること

21．研究成果の公表について

22．知的財産権について

　　　本研究は知的財産権の発生しない研究であるため該当しない。

15.1研究対象者等及びその関係者からの相談等への対応

麻酔担当医師や研究責任者への相談があった場合には他の研究者等の個人情報や、研究者の知的財産権の保護などの回答できない部分を除いて、開示できる情報は状況に応じて適宜提供する。

16. 試料・情報の保管及び廃棄の方法

研究の同意書、登録票、患者および研究データ収集用紙などの紙媒体はそれぞれの種類に分別してファイルしたうえで、施錠が可能なロッカーに保管する。電子媒体の情報は、保管期間満了までログインにパスワードが必要なパソコン内に保管し、パソコンは施錠した室内・ロッカーに保管する。また紙・電子情報は、研究終了後、研究の検証の観点から研究終了後5年もしくは最終結果公表後3年のどちらか遅い時期まで保管した後にデータを完全に消去するという方法ですみやかに廃棄処分する。

17. 研究の資金源等、研究機関の研究に係る利益相反及び個人の収益等、研究者等の研究に係る利益相反に関する状況

17.1 資金源及び財政上の関係

本研究の研究機器（EV1000 ）はエドワーズライフサイエンス株式会社から横浜市立大学附属病院が有償でレンタルする。その他の消耗品費ついては横浜市立大学麻酔科学教室の基礎研究費を用いる。本研究は医学的な視点から行われ、特定の企業・団体の利益や便宜をはかるものではない。

17.2臨床研究に関する費用

研究対象者等に経済的負担又は謝礼がある場合には、その旨及びその内容

本研究に関する医療費は全て通常の健康保険の範囲内で行われ、研究期間中の観察・検査、使用薬剤等にかかる費用の被験者の自己負担分は被験者が支払うものとする。

17.3健康被害に対する補償

本研究は、すでに厚生労働省により承認された医療機器を使用する研究である。そのため、この治療が原因で健康被害が生じた場合については、本研究の担当医師が責任をもって治療にあたるが、その治療費は通常の保険診療による支払いとなる。健康被害に対する金銭的な補償はない。

18. 研究計画書の改変更

研究代表者は必要に応じて、研究実施計画書（プロトコル）を変更する。変更内容の重大性にかかわらず、変更したプロトコル及びその改訂履歴（改訂内容とその理由）を文書で作成し、所属する施設の長及び各参加施設の研究責任医師に報告する。

1. 重大な変更（改正）の場合

倫理委員会へ審議を依頼し、承認を得る。重大性の判断基準として、研究の科学性に関係することと被験者のリスクを増大させることを考慮する必要がある。例としては、適格基準、治療計画、評価項目、予期される有害事象等に関係する変更が挙げられる。

1. 重大でない変更（改訂）の場合

計画にかかわった主たる研究者の承認と倫理委員会への報告を必要とする。審査承認については、各施設の取り決めに従う。本研究に参加する被験者の危険を増大させる可能性がなく、かつ本研究の主要評価項目にも関連しないプロトコルの変更を指す。

19. 研究機関の長への報告内容及び方法

19.1研究の経過報告

　研究の経過報告を年1回、実施状況報告書に記載し報告する。

19.2研究の終了

研究が終了したとき、研究代表者は、研究機関の長にその旨および研究結果の概要を文書で報告する。

19.3研究の早期中止

研究が中止または中断された場合、理由の如何を問わず、実施施設の長に文書で通知する。

1. 安全性に対する問題が明らかとなった場合、または研究継続の意義が無くなったと判断された場合。
2. 症例登録の著しい遅れ、プロトコル逸脱の頻発などの理由により、研究の完遂が困難と判断された場合。

20. 記録の保存

研究代表者は、本研究に関する文書および記録（倫理委員会の記録、同意文書など）を研究代表者の責任において研究終了から5年/最終結果公表の3年のうち遅い方までの間、施錠可能なロッカーに保管し厳重に管理する。

21. 研究に関する情報公開の方法

本臨床研究の終了後、速やかに論文発表または学会での発表などを行う。筆頭著者は研究代表者とする。共著者は研究代表者と研究責任医師の話し合いで決定する。

22. モニタリング及び監査

(指針第20 の規定によるモニタリング及び監査を実施する場合には、その実施体制及び実施手順)

該当しない

23. 研究の実施体制

23.1研究代表者

（所属）麻酔科 　（職名）助教 　（氏名）横瀬　真志

23.2研究事務局

特になし

23.3参加施設および施設の研究責任者

　　本研究は単施設研究である。

23.4 委託業務の内容及び委託先の監督方法

該当なし

23.5　臨床統計家

　　該当なし

23.6症例登録割付およびデータマネジメント

該当なし

# 24．文献

1. Martina JR, et al. Noninvasive continuous arterial blood pressure monitoring with Nexfin®. Anesthesiology. 2012. 116:1092-103.
2. Ameloot K, et al. Validation study of Nexfin® continuous non-invasive blood pressure monitoring in critically ill adult patients. Minerva Anestesiol. 2014. 80:1294-301.
3. Fischer MO, et al. Non-invasive continuous arterial pressure and cardiac index monitoring with Nexfin after cardiac surgery. Br J Anaesth. 2012. 109:514-21.
4. Hofhuizen C, et al. Validation of noninvasive pulse contour cardiac output using finger arterial pressure in cardiac surgery patients requiring fluid therapy. J Crit Care. 2014. 29:161-5.
5. Hohn A, et. Al. Non-invasive continuous arterial pressure monitoring with Nexfin does not sufficiently replace invasive measurements in critically ill patients. Br J Anaesth. 2013. 111:178-84.
6. Monnet X, et al. The estimation of cardiac output by the Nexfin device is of poor reliability for tracking the effects of a fluid challenge. Crit Care. 2012. 16:R212.
7. Stover JF, et al. Noninvasive cardiac output and blood pressure monitoring cannot replace an invasive monitoring system in critically ill patients. BMC Anesthesiol. 2009 ;9:6.
8. Lima AP, et al. Use of a peripheral perfusion index derived from the pulse oximetry signal as a noninvasive indicator of perfusion. Critical Care Medicine 2002. 30: 1210-3.
9. Kim S-H, Lilot M, Sidhu KS, Rinehart J, Yu Z, Canales C, et al. Accuracy and precision of continuous noninvasive arterial pressure monitoring compared with invasive arterial pressure: a systematic review and meta-analysis. Anesthesiology. 2014;120: 1080-1097.
10. Smolle KH, et al. The Accuracy of the CNAP® Device Compared with Invasive Radial Artery Measurements for Providing Continuous Noninvasive Arterial Blood Pressure Readings at a Medical Intensive Care Unit: A Method-Comparison Study. Anesth Analg. 2015. 121:1508-16.

# 25．付録

研究対象機器の添付文書

クリアサイトフィンガーカフ

<http://edwards.jp/jp/professionals/ifus/clearsight>

EV1000

<http://edwards.jp/jp/professionals/ifus/ev1000/>

FloTracセンサー

http://edwards.jp/jp/professionals/ifus/flotrac/
