# Supplementary material for: The perfusion index measured by the pulse oximeter affects the agreement between ClearSight and the arterial catheter-based blood pressures: A prospective observational study
Source: PLoS One. 2019 Jul 10;14(7):e0219511. doi: 10.1371/journal.pone.0219511 (PMC6619788; doi:10.1371/journal.pone.0219511)
Supplement: S1 Table — (DOCX) [file pone.0219511.s005.docx]

**S1 Table.** **The Results of the Linear Mixed-effects Regression Model for the Mean Difference between Invasive Arterial Pressure and Non-invasive ClearSight Arterial Pressure Measurements**

|  | **Systolic arterial pressure** | | **Diastolic arterial pressure** | | **Mean arterial pressure** | |
| --- | --- | --- | --- | --- | --- | --- |
|  | **Adjusted MD (95% CI)** | **P-value** | **Adjusted MD (95% CI)** | **P-value** | **Adjusted MD (95% CI)** | **P-value** |
| PI (>1) | 3.6 (3.0 to 4.2) | <0.001 | -2.3 (-2.7 to -2.0) | <0.001 | -0.3 (-0.7 to 0.1) | 0.20 |
| HR (/10 bmp) | -1.1 (-1.4 to -0.8) | <0.001 | 0 (-0.2 to 0.1) | 0.49 | 0.4 (0.2 to 0.6) | 0.12 |
| mAP (/10 mmHg) | 0.0 (-0.2 to 0.1) | 0.43 | 0.2 (0.0 to 0.4) | 0.02 | -0.4 (-0.5 to -0.2) | <0.001 |
| T core (/1 degree) | -1.5 (-1.9 to -1.1) | <0.001 | -0.6 (-0.8 to -0.4) | <0.001 | -0.8 (-1.0 to -0.5) | <0.001 |

HR, heart rate; mAP, mean of arterial pressures, (systolic arterial pressure measured by ClearSight + invasive systolic arterial pressure) / 2, calculated similarly for the diastolic and mean arterial pressure; MD, mean difference of bias; PI, perfusion index; T core, bladder temperature of patients.
